# Ancestral state reconstruction of metabolic pathways across pangenome ensembles

**DOI:** 10.1099/mgen.0.000429

**Published:** 2020-09-14

**Authors:** Fotis E. Psomopoulos, Jacques van Helden, Claudine Médigue, Anastasia Chasapi, Christos A. Ouzounis

**Affiliations:** ^1^​ Institute of Applied Biosciences (INAB), Center for Research & Technology Hellas (CERTH), GR-57001 Thessalonica, Greece; ^2^​ Lab. Technological Advances for Genomics & Clinics (TAGC), Université d'Aix-Marseille (AMU), INSERM Unit U1090, 163, Avenue de Luminy, 13288 Marseille cedex 09, France; ^3^​ UMR 8030, CNRS, Université Evry-Val-d'Essonne, CEA, Institut de Biologie François Jacob – Genoscope, Laboratoire d'Analyses Bioinformatiques pour la Génomique et le Métabolisme, Evry, France; ^4^​ Biological Computation & Process Laboratory (BCPL), Chemical Process & Energy Resources Institute (CPERI), Center for Research & Technology Hellas (CERTH), GR-57001 Thessalonica, Greece

**Keywords:** comparative genomics, metabolic pathways, ancestral reconstruction, parsimony method, phylogenetic profiling

## Abstract

As genome sequencing efforts are unveiling the genetic diversity of the biosphere with an unprecedented speed, there is a need to accurately describe the structural and functional properties of groups of extant species whose genomes have been sequenced, as well as their inferred ancestors, at any given taxonomic level of their phylogeny. Elaborate approaches for the reconstruction of ancestral states at the sequence level have been developed, subsequently augmented by methods based on gene content. While these approaches of sequence or gene-content reconstruction have been successfully deployed, there has been less progress on the explicit inference of functional properties of ancestral genomes, in terms of metabolic pathways and other cellular processes. Herein, we describe PathTrace, an efficient algorithm for parsimony-based reconstructions of the evolutionary history of individual metabolic pathways, pivotal representations of key functional modules of cellular function. The algorithm is implemented as a five-step process through which pathways are represented as fuzzy vectors, where each enzyme is associated with a taxonomic conservation value derived from the phylogenetic profile of its protein sequence. The method is evaluated with a selected benchmark set of pathways against collections of genome sequences from key data resources. By deploying a pangenome-driven approach for pathway sets, we demonstrate that the inferred patterns are largely insensitive to noise, as opposed to gene-content reconstruction methods. In addition, the resulting reconstructions are closely correlated with the evolutionary distance of the taxa under study, suggesting that a diligent selection of target pangenomes is essential for maintaining cohesiveness of the method and consistency of the inference, serving as an internal control for an arbitrary selection of queries. The PathTrace method is a first step towards the large-scale analysis of metabolic pathway evolution and our deeper understanding of functional relationships reflected in emerging pangenome collections.

## Data Summary

We provide the entire PathTrace module written in Perl and Java, and appropriately documented along with sample input data for experimentation by the community through the GitHub repository at https://github.com/CGU-CERTH/PathTrace under an MIT license. Query enzyme sequences, target pangenome ensembles and similarity search results are available on Figshare (https://figshare.com/); specific links are provided at the GitHub directory listed above.

**Impact Statement**

Metabolic pathways represent an elegant formulation of functional units for cell physiology, whose efficacy relies on the combined activities of enzymes catalysing a set of interconnected reactions. As in any phylogeny, pathways reflect patterns of evolutionary events, leading to stability, loss or gain – observed as intact sets, fragmented remnants or augmented decorations, of the corresponding enzyme groups, respectively. With multiple genomic sequences at hand, methods that allow the inference of ancestral states for genes or genomes have been developed. In order to bridge these two ultimate aspects of evolutionary change, i.e. gene-sequence evolution and genome phylogeny, the inference of ancestral metabolic pathways offers a fresh view that focuses on genome function and physiology, explaining how pathway presence or absence reflects the functional capabilities of particular species. In this work, we describe a parsimonious method based on the ancestral inference of gene content, using metabolic pathways and entire pangenomes, collections of genomic sequences across taxonomic groups. We provide examples of how pangenomes contribute to the accurate detection of pathways and how pathways are inherited, lost or gained across a phylogeny of distantly related taxa. Our approach forms a basis upon which metabolic pathways can be understood in the context of pangenome information, in an automated, highly scalable fashion for large-scale computations of ancestral inference.

## Introduction

The investigation of evolutionary histories of characters in terms of structure and function lies at the heart of biological research [1]. With the advent of genomic revolution, much of this work was performed at the genomic sequence or even at the protein-sequence level, focusing on specific patterns of phylogeny or protein function [2, 3], all the way to practical applications for synthetic biology [4].

Earlier approaches are mainly based on gene content or protein-sequence reconstructions [5–8]. Phylogenetic profiles and their various incarnations coupled with sophisticated simulation experiments have been extensively used, due to their inherent ability to summarize evolutionary patterns in an elegant, efficient and succinct fashion [9, 10]. Among the available algorithms, GeneTrace [11] represents one of the first scalable approaches that performed genome-wide ancestral gene-content inference on a large scale [12]. In this wider, collective effort, a recurring theme has always been the identification and further validation of lateral gene transfers [13–15], which present significant challenges to the underlying evolutionary models [16, 17].

Shifting away from the structural interpretation of genes, proteins and protein families – typically captured by simple profiles of gene content, it is desirable to formulate additional models that address the presence, emergence, loss or transfer of entire cellular processes, represented by enzyme groups in their corresponding metabolic pathways. Work along these lines has addressed the parsimonious inference of pathways in (meta)/genomes [18] or changes in protein interaction networks [19], while other studies have aimed at the estimation of gene gain and loss using Bayesian models [20]. With respect to metabolic pathways, one of the earliest, most prominent advances on the subject has explored the varying degree of pathway evolution, with abrupt patterns of gain and loss – facilitated by massive lateral gene transfers [21, 22]. Other directions that have been explored – not directly comparable with the inference of ancestral states per se – are represented by CLIME, which attempts to expand the initial pathway query and discover additional links based on phylogenetic profiles [23], MinPath, which detects pathway overpredictions in genomic and metagenomic datasets [18] and CoReCo, which estimates ‘extant’ (present) and ‘extinct’ (ancestral, i.e. inferred) genome-scale metabolic models [24].

Here we describe a robust, efficient and scalable algorithm, named PathTrace, that addresses the issue of pathway inference across multiple ancestral genomes on a phylogeny for arbitrarily chosen target datasets. Our results indicate that pangenomes form the basis upon which an elaborate query scheme can yield meaningful insights, with high accuracy. In addition, visualization of ancestral inference results can be facilitated by BioPAXViz, a Cytoscape plugin [25] that allows the browsing of single pathway histories or comparison of multiple pathways [26].

## Methods

In order to infer the evolutionary history of a metabolic pathway, it is essential to project the enzyme sequences involved in that pathway onto a species phylogeny, including all ancestral nodes. We present a method called PathTrace that allows the inference of the most parsimonious evolutionary scenario that might have led to the most likely, observed present-day instance of a metabolic pathway. The PathTrace input consists of a metabolic pathway entry and an evolutionary tree that covers all organisms under consideration. Inner nodes of the tree represent ancestral organisms. Conversion from Newick phylogenetic tree format is provided by the utility newick2nodes.pl in the GeneTrace directory of the distribution.

**Fig. 1.**
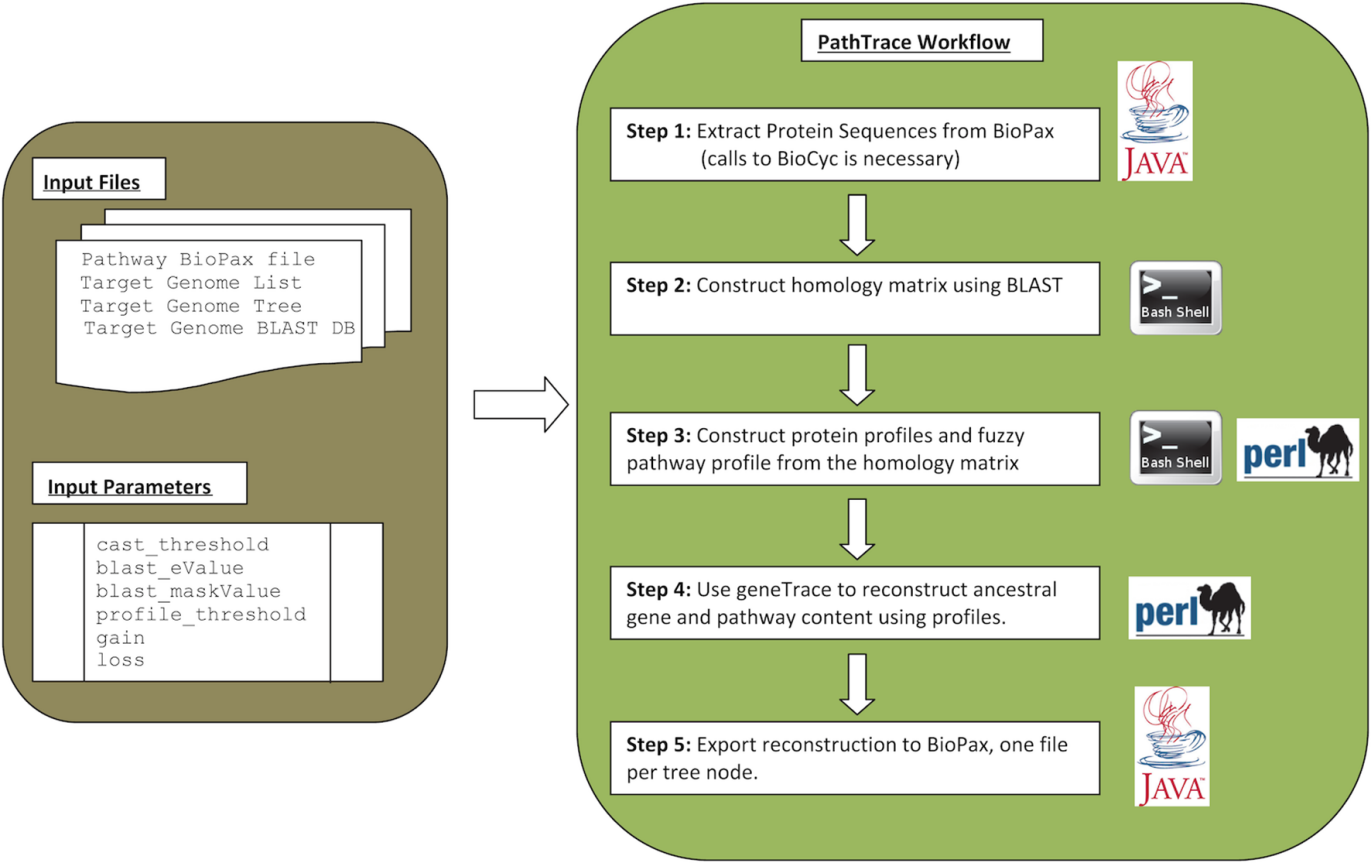
Overview of the main steps of PathTrace, including input, output and parameter set. The PathTrace method encompasses three main computational steps, surrounded by two steps for input/output. During the input phase, the algorithm extracts the protein sequences from the provided BioPAX files, through direct API calls to BioCyc. The next three steps include the construction of both the homology matrix and the corresponding fuzzy pathway profiles, leading to the reconstruction of the gene and pathway content, using GeneTrace. Finally, the produced output is bundled as a set of BioPAX-formated files, one for each node of the corresponding tree.

The process is shown in Fig. 1, and is described in more detail below, in five distinct, sequential steps.

► Step 1: extract protein sequences from **BioPAX** files.

The metabolic pathway under study is represented by a BioPAX file [27]. However, the content of any given BioPAX file may vary significantly. This step addresses the issue of extracting protein product sequence information either directly from the file or, if not available, through an online connection to a public database such as BioCyc [28] or KEGG [29], among others – see also http://www.biopax.org/mediawiki/index.php/Data.

► Step 2: homology matrix construction.

Using the protein sequences of the enzymes from the query pathway at the first step, we construct a homology matrix, i.e. gene-content vectors, whose elements represent the number of homologues of genes belonging to each target genome.

In formal notation,

$$V_{HG_{i}}\;=[h_{g_{i}}^{1}\;\;h_{g_{i}}^{2}\cdot \cdot \cdot \;h_{g_{i}}^{m}]\;and\;M_{H}\;=\left[\begin{array}{c} V_{HG_{1}}\\ V_{HG_{2}}\\ \vdots \\ V_{HG_{n}} \end{array}\right]$$

where $V_{HG_{i}}$ is the homology vector of protein *g*_*i*_ across *m* target genomes, $h_{g_{i}}^{j}$ is the count of homologues of protein *g*_*i*_ existing in genome *j*, and *M*_H_ is the homology matrix composed of the homology vectors of the *n* protein products participating in the pathway under investigation.

► Step 3: from homology matrix to fuzzy pathway profiles.

The key concept for any evolutionary method with regard to metabolic pathway evolution is the projection of comparative genomics information (e.g. gene content) onto pathway patterns. Our approach is similar to fuzzy phylogenetic profiles [30], as it attempts to represent the likelihood of the presence of a pathway across the target genomes. Presence needs to be quantified as a ‘fuzzy presence’, i.e. a measure of likelihood that the pathway is present in a species: this quantity may range from zero for complete absence, to partial presence, or in fact complete presence – the latter are represented by unbound real numbers.

For a given pathway with known protein products, the formal notation of a fuzzy pathway profile is the following:

$$P_{f}\;=[f_{1}\;f_{2}\cdot \cdot \cdot f_{m}]\;=\left[\frac{\sum_{i=1}^{n}h_{g_{i}}^{1}}{n}\frac{\sum_{i=1}^{n}h_{g_{i}}^{2}}{n}\cdot \cdot \cdot \frac{\sum_{i=1}^{n}h_{g_{i}}^{m}}{n}\right]$$

where *P*_*f*_ is the fuzzy profile of a pathway and *f*_*j*_ is the fuzzy presence of the pathway in genome *j*. Notation follows step 2, i.e. $h_{g_{i}}^{j}$ is the count of homologues of protein *g*_*i*_ existing in genome *j*.

The fuzzy-pathway profile formulation captures the available variance of the presence/absence of a pathway across a given set of target genomes in a composite fashion. Furthermore, it is critical to establish an accurate baseline of presence/absence, therefore the fuzzy real-value profile needs to be reformulated into a discrete binary vector. The discretization process requires an additional parameter *α*, which sets the boundary of presence for a corresponding threshold of homologues. The final, discrete, pathway profile in formal notation is as follows:

$$P_{d}\;=[d_{1}\;d_{1}\cdot \cdot \cdot d_{m}]\;where\;d_{j}\;=\left\{\begin{array}{c} 0,\;if\;f_{j}§lt;a\\ 1,\;if\;f_{j}\geq a \end{array}\right\}$$

It is important to note at this point that the discretization threshold *α* is directly affected by the evolutionary distances within the species group under study. An intuitive explanation towards this point is that closely related species would exhibit highly similar pathways and gene content, and thus a more restrictive threshold is required to showcase the differences between them. On the other hand, evolutionarily distant species would exhibit large variations and thus a low, permissive threshold would identify the most critical changes among them (see also Results).

► Step 4: ancestral pathway reconstruction.

The next step in the PathTrace process is to utilize the discrete pathway profiles, in order to assign pathway presence or absence at each node of the evolutionary tree, provided as input. In order to better define this step, we use the definition of the tree structure *T* as an ordered pair (*V*, *E*) comprising a set of nodes *V* and a set of edges *E*, where each edge is a two-element subset of *V*, i.e. *E* = (*v*_*i*_, *v*_*j*_) where *v*_*i*_, *v*_*j*_∈ *V*. Moreover, a tree structure must conform to the constraint that *T* must be a connected graph and *E* = |*V* □ 1. A key attribute of *T* is that for an edge (*u*, *v*) in *T*, *u* is the parent of *v*, and *v* is a child of *u*, i.e. all edges are directional. Using this attribute, we can identify the following special cases:

- if *u* has children, then *u* is internal;
- if *u* has no children, then *u* is a leaf;
- if *v* has no parent, then *v* is the root of *T*;
- *u* and *v* are siblings if they have same parent.

Finally, given two nodes *v*_*i*_, *v*_*j*_ in *T*, if there is a path from *v*_*i*_ to *v*_*j*_ then *v*_*i*_ is an ancestor of *v*_*j*_, *v*_*j*_ is a descendent of *v*_*i*_, and *v*_*j*_ is in *v*_*i*_’s subtree, i.e. *v*_*j*_∈ *T*(*v*_*i*_).

Within the context of the PathTrace process, we need to identify each node of the tree with a value. Without loss of generality, let each node *v* contain a single attribute *p* ∈ {TRUE,FALSE} that denotes presence [*p*(*v*)=TRUE] or absence [*p*(*v*)=FALSE of the pathway under consideration at that particular node.

The PathTrace process takes a bottom-up approach for the characterization of the nodes in *T*, beginning at the leaf level and pushing up to the root of the tree. It is important to note that, in the case of an evolutionary tree, the leaf nodes correspond to the current range of ‘extant’ genomes (i.e. the target genome set in PathTrace notation), whereas all internal nodes, as well as the root of *T*, correspond to the ‘extinct’ ancestral nodes. The algorithm underlying this step is GeneTrace [11], meaning that the assignment conforms to the following cases:

1. Given a parent node *u* and all respective *k* children nodes *v*_*i*_, then *p*(*u*)=*p*(*v*_*i*_), ∀ *v*_*i*_ ∈ {(*u*, *v*_*i*_), *i*=1.*k*} and *p*(*v*_1_)=*p*(*v*_2_) = …=*p*(*v*_*k*_). Simply put, a parent node retains the pattern of the children nodes only in case the latter exhibit a uniform pattern, either presence or absence.
2. Given a parent node *u* and all respective *k* children nodes *v*_*i*_, then if ∃ *v*_*j*_ : *p*(*v*_*j*_) ≠ *p*(*v*_*i*_), ∀ *v*_*i*_ ∈ {(*u*, *v*_*i*_), *i*=1.*k*}, *v*_*i*_, *p*(*v*_*i*_)=TRUE -|*v*_*j*_, *p*(*v*_*j*_)=FALSE >GAIN and ∃ *v*_*i*_, *v*_*j*_∈ {(*u*, *v*_*i*_),*i*=1.*k*}: *p*[TRUE(*v*_*i*_)]=*p*[TRUE(*v*_*j*_)]=TRUE, then *p*(*u*)=TRUE. Simply put, a parent node is assigned to pathway presence if the difference between the number of potential gains and losses is larger than a threshold value GAIN, and pathway presence is detected in at least two children subtrees.
3. Given a parent node *u* and all respective *k* children nodes *v*_*i*_, then if ∃ *v*_*j*_ : *p*(*v*_*j*_) ≠ *p*(*v*_*i*_), ∀ *v*_*i*_ ∈ {(*u*, *v*_*i*_), *i*=1.*k*}, *v*_*i*_, *p*(*v*_*i*_)=FALSE -|*v*_*j*_, *p*(*v*_*j*_)=TRUE >LOSS, then *p*(*u*)=FALSE. Simply put, a parent node is assigned to pathway absence if the difference between the number of potential gains and losses is larger than a threshold value LOSS.

Finally, it is important to note that, in contrast to the GeneTrace algorithm, the ancestral pathway reconstruction process is largely insensitive with regards to changes in the GAIN and LOSS thresholds, due to the composite and thus robust nature of pathway profiles.

► Step 5: output to **BioPAX** and visualization.

The final step of the PathTrace method is the output of the reconstruction information into a computer-readable and universal format. To this end, we have selected the BioPAX file format as the most suitable choice [27].

### Definition of presence/absence in BioPAX format

We have extended the BioPAX core functionality with the addition of a flag within a BioPAX file, indicating the presence or absence of an entire pathway or of specific proteins of the pathway. The BioPAX ontology representation is OWL-based and, in this sense, pathways are represented as ‘Pathway’ xml-elements while proteins are represented as ‘ProteinReference’ xml-elements within a BioPAX file [27]. Both of these BioPAX elements share some common attributes and also possess some additional ones, depending on the functionality of the biological entity they describe. However, the BioPAX L3 format release does not provide attributes that could designate via a flag the presence or absence of a BioPAX element in a BioPAX file. To this end, and in order to avoid ambiguity and potentially erroneous interpretation of the data within a BioPAX file, we decided to insert the presence/absence flag through a custom structured comment. The custom comment should start with a predefined prefix, as a sub-element of a ‘Pathway’ or a ‘ProteinReference’ element in a BioPAX file. Specifically, the prefix of the custom comment we have introduced is ‘$custom comment$:’ (without the quotes) and it is followed by the word ‘present’ or ‘absent’ (without any spaces) in case the protein/pathway is present or absent, respectively.

For example, if a ProteinReference instance (namely ‘protRefId1’) is absent from a pathway then the corresponding ‘ProteinReference’ element in the BioPAX file will have the following form:

**Table:** 

<bp:ProteinReference rdf:about=“protRefId1”> <bp:comment rdf:datatype=“http://www.w3.org/2001/XMLSchema#string”>$custom comment$:absent</bp:comment> </bp:ProteinReference>

Finally, the aforementioned BioPAX functionality extension provides us with the possibility of both creating alternative versions of pathways where some or all of the nodes are missing, and additionally highlighting the presence or absence of entire pathways from specific organisms.

### Data resources and algorithms

Development and analysis were performed using both sequence and metabolic pathway information.

In order to acquire high-quality, annotated protein sequences, we selected the entire Bacteria EnsemblGenomes database (http://bacteria.ensembl.org/, release 12), from which we retrieved a total of 937 529 protein sequences from 249 entries, organized into ten major collections (original data available in URL ftp://ftp.ensemblgenomes.org/pub/bacteria/release-12). The ten collections provided by EnsemblGenomes, namely Mycobacterium, Streptococcus, Bacillus, Staphylococcus, Wolbachia, Buchnera, Escherichia/Shigella, Neisseria and Borrelia provide an extensive coverage of the domain Bacteria, while the collection for Pyrococcus represents an – arbitrarily chosen – representative from the domain Archaea, serving as an ougroup for phylogenetic tree construction (Table 1). The choice of an early release (2012) was dictated by the project design, collaborative annotation, and proper interpretation; it does not imply lack of scalability, which is limited by Step 2 (homology matrix construction). Other search algorithms that speed up similarity searches might be considered in the future (see below). The design of PathTrace allows the execution of other algorithms, such as DIAMOND [31], provided that the input format is transformed to that of the current version.

**Table 1.** Ten pangenome collections used in this study as target species, at the genus level; ‘block number’ signifies the partitioning of the phylogenetic profiles in the relevant figures (see below). For details, see also Table S1 (available in the online version of this article). Total number of genomes is 249 (used in Results i-ii); 182 of those have been selected for validation (used in Results iii, marked by an ^*^asterisk, listed in Table 3)

Block no.	Genus (pangenome)	No. of genomes
1	* Pyrococcus* spp.	4^*^
2	* Mycobacterium* spp.	20
3	* Streptococcus* spp.	48^*^
4	* Bacillus* spp.	78^*^
5	* Staphylococcus* spp.	27
6	* Wolbachia* spp.	4
7	* Buchnera* spp.	6^*^
8	*Escherichia/Shigella* spp.	46^*^
9	* Neisseria* spp.	8
10	* Borrelia* spp.	8

For the case study involving a large-scale experiment (see Results), nine pathways were defined as queries (Table 2) against a selection of a single pangenome for five genera coupled with genome instances of two strains from each genus (Table 3). In total, 182 single genomes were thus defined as a target set, with sufficient taxonomic coverage and full pangenome information. As a recommendation for users, the target set has to satisfy the criteria for the definition of a pangenome, for instance the saturation curves for close pangenomes or the bimodal frequency distribution of protein families [32]. These simple and reasonable criteria have to be respected before a target set is built for querying by pathway sets, perhaps with the exception of including an outgroup, as in the present work (see below).

**Table 2.** The nine metabolic pathways obtained from BioCyc as queries. Column ‘#Enzymes’ corresponds to the number of unique protein sequences for the enzymes of each pathway – no homologues or any other protein family information has been extracted. Source genome lists the three species/strains from which the pathways have been extracted. The Glycolysis V (* Pyrococcus *, an archaeal species) has been selected as an outgroup. Total number of enzymes is 86

	Pathway name	#Enzymes	Source genome
1	Leucine Biosynthesis I	6	* Escherichia coli* K-12 substr. MG1655
2	TCA cycle I (prokaryotic)	18	* Escherichia coli* K-12 substr. MG1655
3	Methionine Biosynthesis I	6	* Escherichia coli* K-12 substr. MG1655
4	Isoleucine Biosynthesis I (from threonine)	11	* Escherichia coli* K-12 substr. MG1655
5	Lysine Biosynthesis I	9	* Escherichia coli* K-12 substr. MG1655
6	Biotin Biosynthesis I	11	* Escherichia coli* K-12 substr. MG1655
7	Glycolysis V (Pyrococcus)	8	* Pyrococcus horikoshii* OT3
8	Lysine Biosynthesis II	12	* Bacillus subtilis* subtilis 168
9	Biotin Biosynthesis II	5	* Bacillus subtilis* subtilis 168

**Table 3.** List of the pangenomes of five genera namely *Escherichia, Buchnera, Bacillus, Streptococcus* and *Pyrococcus* used as the target set with maximum taxonomic coverage, using the nine pathway queries (see Methods). Internal codes in Cogent style [70] are provided (see Table S1)

	Genome name	Internal code
1	* Escherichia coli* K12	ECOL-K12
2	* Escherichia coli* DH10B	ECOL-DH1
3	* Escherichia coli* Pangenome	ECOL-png
4	* Buchnera aphidicola* Schizaphis	BAPH-SCH
5	* Buchnera aphidicola* 5a	BAPH-5AX
6	* Buchnera* Pangenome	BUCH-png
7	* Bacillus subtilis *	BSUB-XXX
8	* Bacillus anthracis* ames ancestor	BANT-AMA
9	* Bacillus* Pangenome	BACI-png
10	* Streptococcus pyogenes* SF370	SPYO-SF3
11	* Streptococcus pneumoniae* 70 585	SPNE-705
12	* Streptococcus* Pangenome	STRE-png
13	*Pyrococcus abyssi*	PABY-XXX
14	* Pyrococcus horikoshii *	PHOR-XXX
15	* Pyrococcus* Pangenome	PYRO-png

Metabolic pathway data for target pathways were retrieved as Level 3 (L3) BioPAX files from the BioCyc database (https://biocyc.org/). The selection of pathways was such that it can provide a wide coverage of representative pathways with multiple enzymes/reactions, including some of the best-characterized cases biochemically.

Similarity searches that are used to detect the number of homologues were performed using BlastP (version 2.2.31, build 7 January 2016) [33] for Unix, using a parameter set as follows (see Fig. 1): CAST threshold 30 with default parameters [34], blast e-value cut-off 10^−06^, no additional masking, PathTrace threshold *α*=1.37 (see below), GeneTrace gain=5/loss=2.

Software development for PathTrace was performed using Perl (version 5.24.0), coupled with the Tree::Simple module and other Unix utilities (such as awk).

Evaluation for comparison purposes with existing tools as well as visual representation of the output, the Mesquite platform was also used [35]. The tool compare-classes from the RSAT/NeAT suite [36] was used for further cross-checks with EC number assignment.

The analysis presented in this work is fully documented, and the relevant input/output files are provided along with the application, to ensure reproducibility and optimal use by the community.

## Results

To establish the PathTrace method and validate results through a number of experiments, we have performed the following analyses; (i) an exploration of the impact that target genome selection might have on the pathway reconstruction process, (ii) a validation of the fuzzy-pathway profile formulation, utilizing an alternative mapping using E.C. numbers, and (iii) an assessment of the sensitivity of the method regarding the GAIN and LOSS parameters. The latter involves a carefully crafted case study using several well-investigated metabolic pathways and the validation of some of the evolutionary scenarios through comparison with the Mesquite suite [35].

### Target genome selection: pangenomes improve accuracy

One of the key issues in any ancestral reconstruction analysis can be the optimal selection of the target genomes, i.e. the selection of the subset of the current genomes against which the reconstruction is performed. The range of options is essentially as follows: (i) several target genomes of varying phylogenetic distance, (ii) a single target pangenome and (iii) several different target pangenomes.

**Fig. 2.**
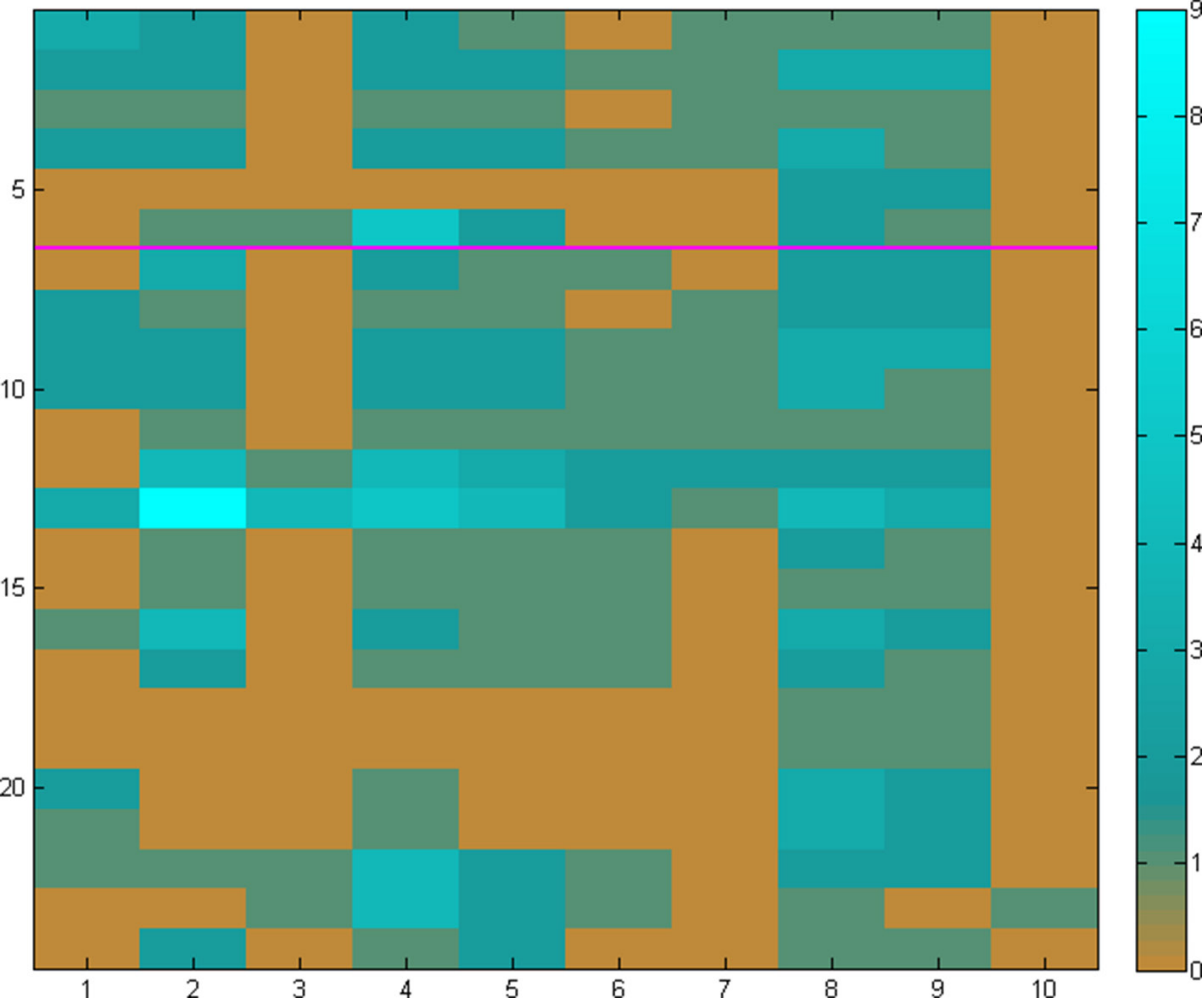
The homology matrix constructed for the 24 protein (enzyme) sequences involved in the Leucine biosynthesis pathway (six enzymes, rows 1–6) and the TCA Cycle (18 enzymes, rows 7–24) – separated by a purple horizontal line, across ten genomes, one from each of the 10 collections used (Table 1). Each row of the matrix corresponds to the profile of a single enzyme, whereas each column corresponds to the homology patterns for a single genome. It is evident that the quasi-random selection of the ten distinct genomes leads to a very heterogeneous form of the matrix, e.g. column 3 (* Streptococcus *) would generally mean absence of both pathways while column 8 (* Escherichia *) would correspondingly indicate presence. In this example, the contrast is too sharp, thus making the decision for pathway presence or absence highly uncertain. Scale from 0 to 9 (the number of homologues, arbitrary value) is shown on the right.

**Fig. 3.**
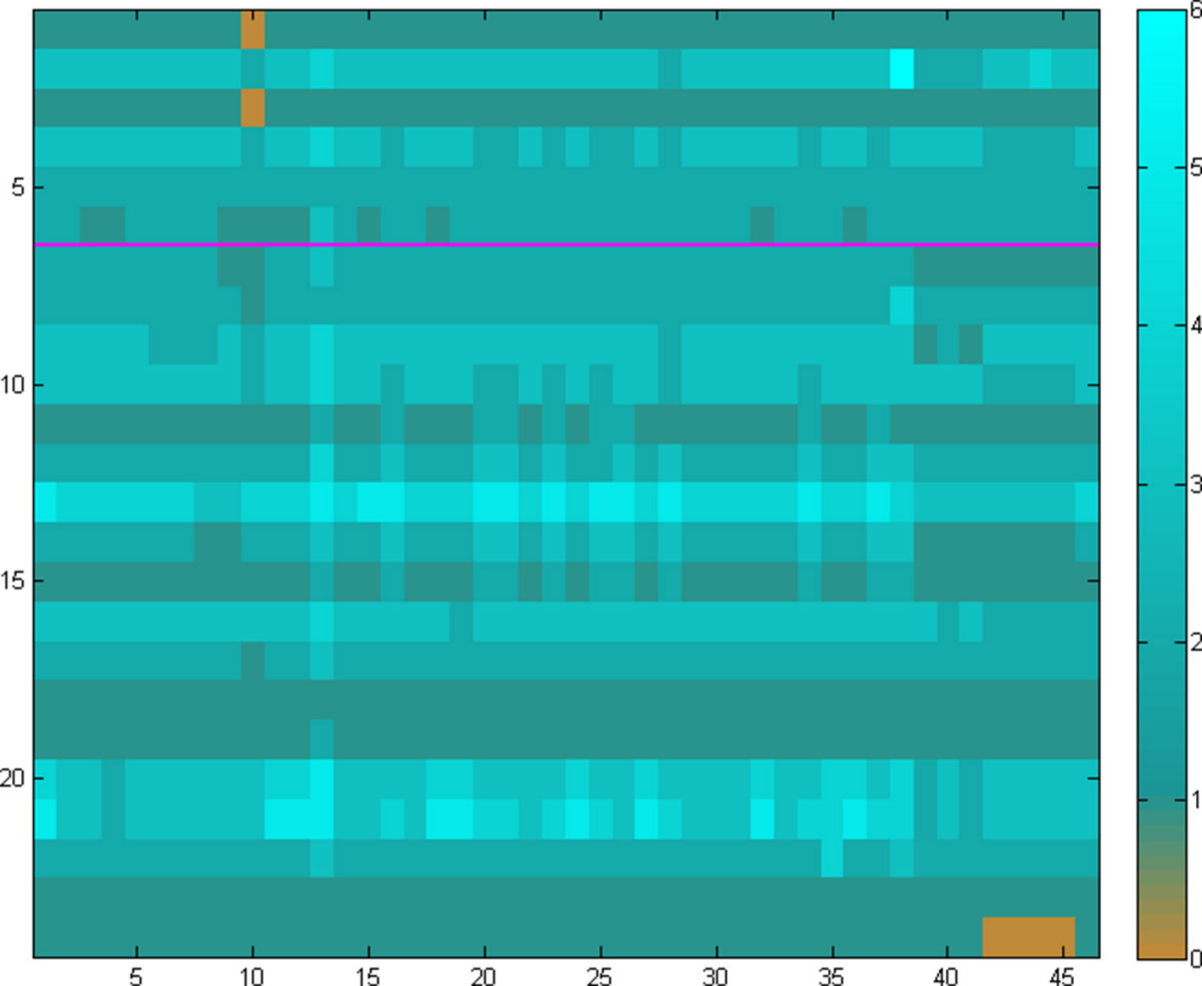
The homology matrix of the same set of 24 enzymes as in Fig. 2 (Leucine biosynthesis in rows 1–6 and TCA Cycle in rows 7–24), using the pangenome approach. In this instance, the columns correspond to the 46 strains present in the entire *Escherichia/Shigella* collection (Table 1). In this case, the crisp choice of a single pangenome of multiple, highly related strains, produces a homogeneous pattern (e.g. presence) of the two pathway examples. Using this strategy, the contrast can be low resulting in a biassed result, thus confounding the assessment of pathway presence or absence, regardless of the accuracy of the phylogeny for the pangenome. Scale from 0 to 6 (the number of homologues, arbitrary value) is shown on the right.

In the first case, the quasi-random choice of distinct genomes in the target set leads to a heterogeneous collection of elements with a broad range, potentially yielding a sharp contrast of phylogenetic profiling, which can be hard to evaluate (Fig. 2). The reverse situation emerges in the case of a single pangenome set (Fig. 3); the choice of a pangenome with a narrow range can lead to a homogeneous collection of elements with little contrast, effectively limiting the ability to evaluate the outcome.

**Fig. 4.**
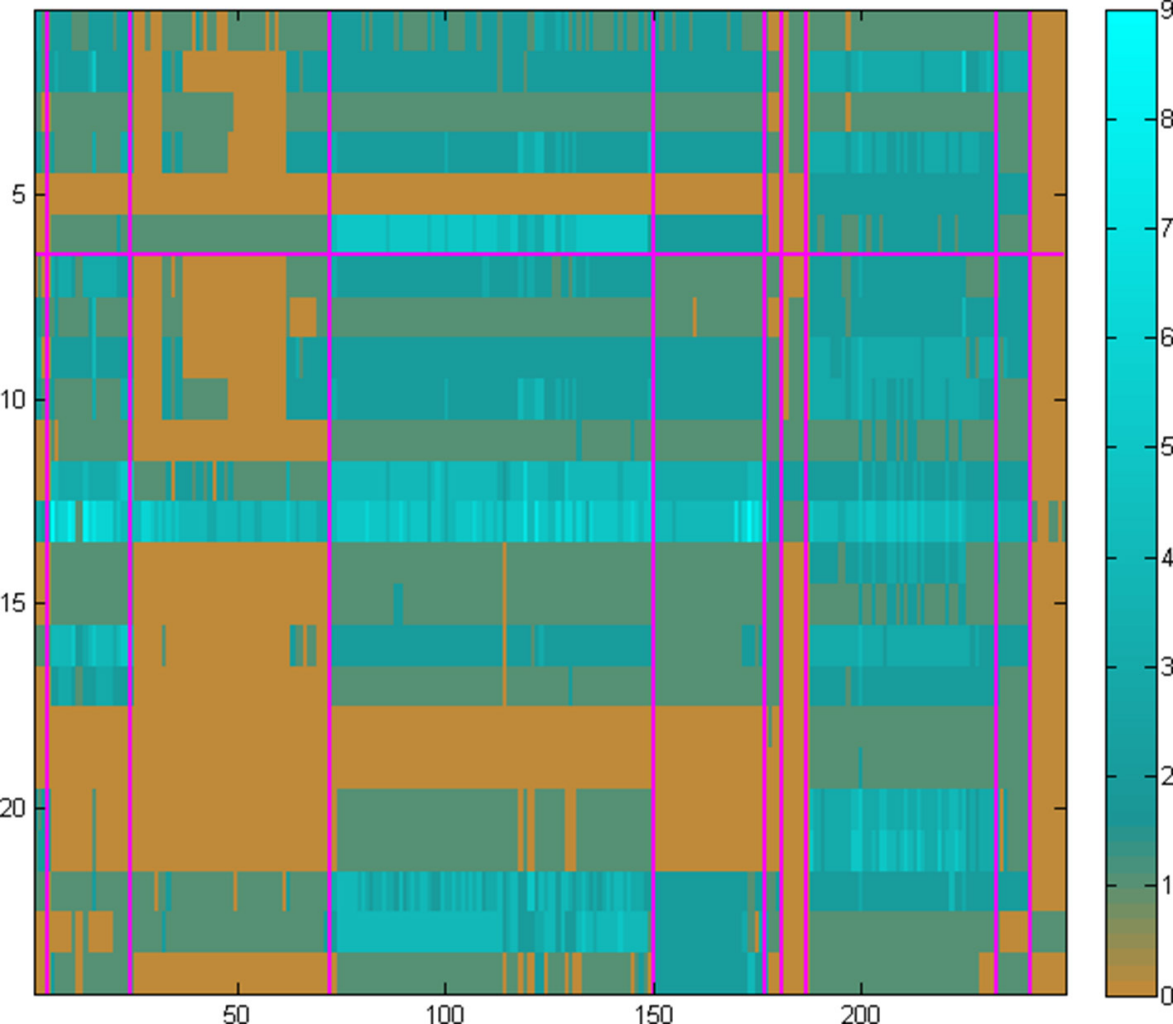
The homology matrix of the same set of 24 enzymes (Leucine biosynthesis in rows 1–6 and TCA Cycle in rows 7–24) – as in Figs 2 and 3, using several pangenomes as the target set. In this instance, the 249 columns correspond to the entire set of available genomes in the Bacteria EnsemblGenomes database (release 12), organized as a set of ten pangenomes (Table 1), outlined by the vertical purple lines. The mixture of representative genomes and pangenomes maintains the required heterogeneity of pathway information with an ‘optimum’ contrast, thus providing evidence of loss (i.e. absence) of reactions across entire pangenomes. This information is consequently used to construct the fuzzy-pathway profiles, even with a coarse-grained phylogeny. Scale from 0 to 9 (the number of homologues, arbitrary value) is shown on the right.

We have learnt, through much experimentation with genome and pangenome ensembles and parameter choices, that an optimal contrast for the target genome set can be achieved through a combination of the previous options in their extremes, i.e. using several pangenomes (Fig. 4). In this case, a mixture of representative pangenomes using all available information maintains the heterogeneity of the target species collection, by providing sufficient resolution and at the same time retaining the evidence of ‘loss’ of reactions (i.e. enzymes) across species and strains, with an optimum pathway contrast.

The above analysis demonstrates that the estimation of pathway presence or absence critically depends on an adequate selection of target genomes. This choice needs to reflect both the conservation of pathway elements in terms of phylogenetic profiles of the corresponding enzyme sequences (typically within a pangenome, i.e. strains) as well as the variation of those elements (typically across genomes, i.e. species). We thus require that any set of genomes selected as a target collection needs to exhibit both high contrast (i.e. entropy) as well as high segmentation (i.e. gradient entropy) – quantified by those parameters, following basic principles from the field of pattern recognition [37]. In our examples above, a random selection of target genomes, with one genus per pangenome (Fig. 2), shows a high level of entropy (0.99) and a low level of segmentation (0.30). In the case of a single pangenome, however rich in the number of strains, exhibits the opposite behaviour (Fig. 3), with low entropy (0.08) and high segmentation (0.82). The optimal solution in this particular case emerges as the one with several pangenomes covering the corresponding genus with multiple species and strains (Fig. 4), with high entropy (0.92) as well as segmentation (0.93). This pattern has been observed consistently across our experiments with a range of pathways and species/strain groups (not shown) and has resulted in the optimal choice of both input datasets and parameter choices during the development of PathTrace. A recommendation is to maintain these two metrics in the resulting homology matrices, as above.

### E.C. numbers: reaction information in agreement with genome comparisons

An alternative approach for the detection of presence or absence of a given pathway against a target genome list, is to utilize reaction information related to that pathway, i.e. the E.C. numbers of the participating enzymes [38]. In order to perform this type of analysis, we have used the RSAT suite and specifically the ‘NeAT – compare-classes’ module [39]. This algorithm accepts as input a list of pairs in the form of {E.C. Number, Genome ID} and a list of pairs in the form of {E.C. Number, Pathway ID} and produces an intersection metric, with a significance level estimated by a hyper-geometric distribution [39].

**Fig. 5.**
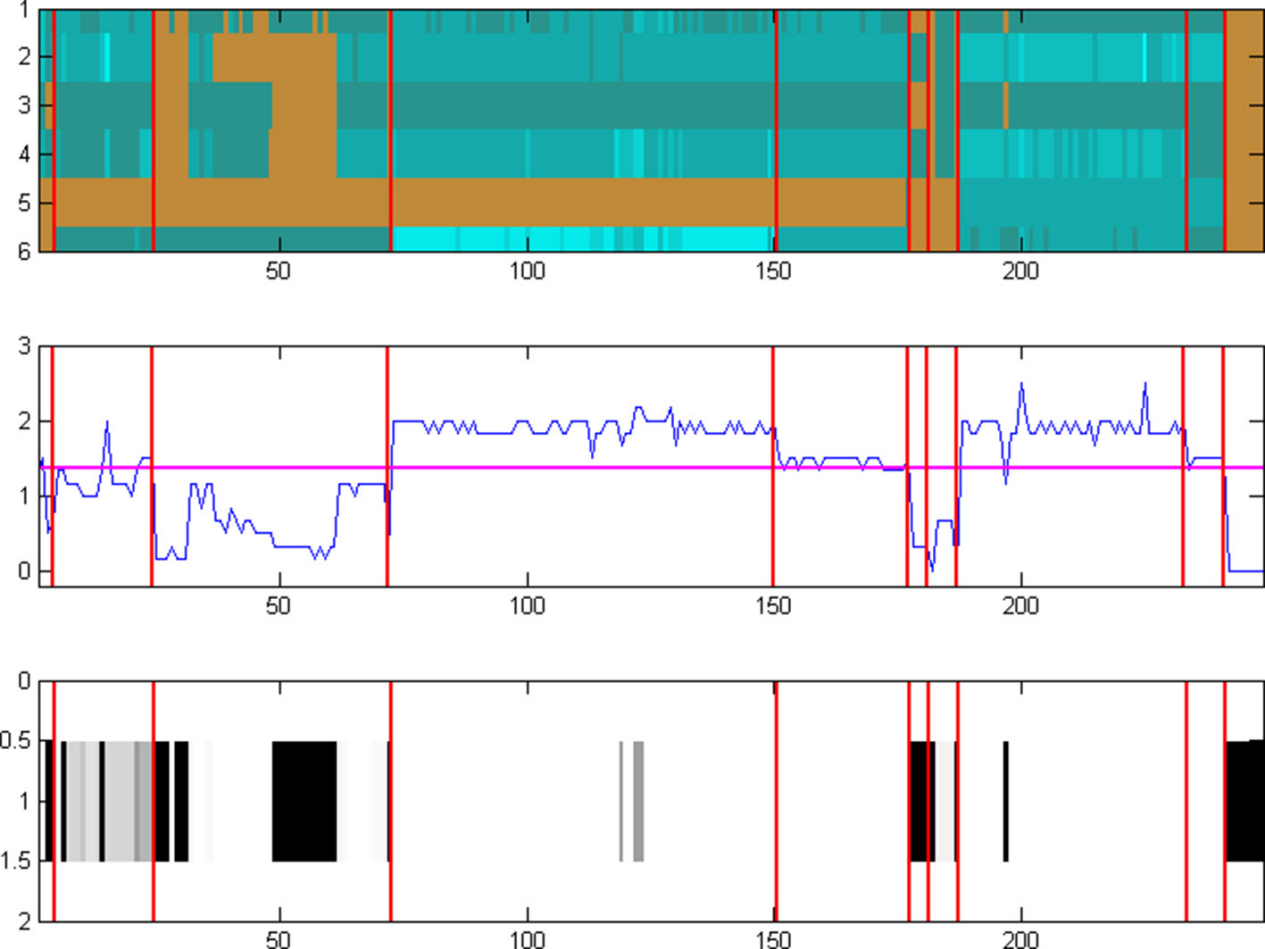
Comparison of three different representations of pathway status (presence/absence), using the Leucine biosynthesis pathway as a case study. The top panel (identical to rows 1–6 of Fig. 4 – including scale) shows the plain homology matrix of the six enzymes involved in the pathway (rows), across the entire complement of the 249 genomes in the Bacteria EnsemblGenomes database (release 12) organized in ten pangenomes (Table 1), outlined with the red vertical lines for all panels. The bottom panel corresponds to the metric produced by NeAT [39], using a greyscale representation of values ranging from white (presence) to black (absence). The middle panel corresponds to the PathTrace representation of fuzzy-pathway profiles, with the horizontal purple line indicating the threshold for presence (above threshold) or absence (below threshold). It is evident that, although all three representations capture the essence of pathway absence or presence across all genomes and pangenomes, the fuzzy-profile approach of PathTrace (middle panel) provides increased sensitivity in the subtle variations within a single pangenome while still capturing the robust classification between absence and presence. See text for details.

An interesting example in the case of leucine biosynthesis for *E. coli* DH10b can be seen (Fig. 5, in column 198), as an indication of strain diversity within the pangenome. DH10B is strictly auxotrophic only for leucine, as low growth rates are not due to the lack of other amino acids [40]. Indeed, the operon leuLABCD is absent compared to the wild-type, ‘causing both sensitivity to nutritional downshifts and slightly lower growth rates’ [40]. This example vividly demonstrates how the detection of loss in different representations of enzyme profiles (Fig. 5) can be validated with existing knowledge from the literature, where appropriate.

**Fig. 6.**
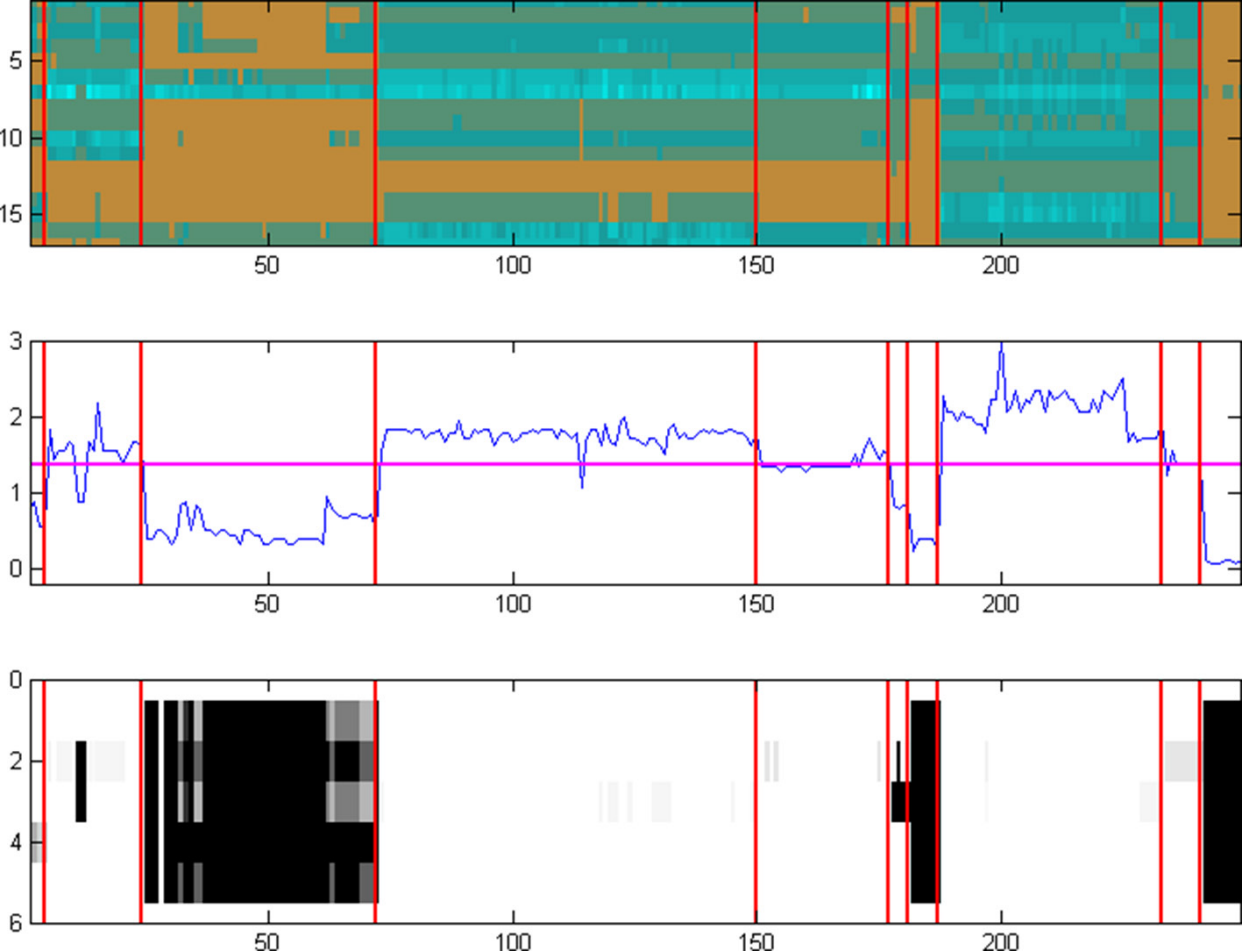
Comparison of three different representations of pathway status (presence/absence), using the TCA Cycle pathway as a case study. The top panel (identical to rows 7–24 of Fig. 4 – including scale) shows the homology matrix of the 18 enzymes involved in the pathway (rows), across the entire complement of the 249 genomes in the Bacteria EnsemblGenomes database (release 12) as organized in ten pangenomes (Table 1). The bottom panel corresponds to the metric produced by NeAT [39] (as in Fig. 5). The middle panel corresponds to the PathTrace representation (as in Fig. 5). Similarly to the Leucine biosynthesis example, subtle patterns of absence or presence across pangenomes can be seen – in this case, Streptococcus (block 3), Buchnera (block 7) and Borrelia (block 10). See text for details.

Evidently the TCA cycle appears to be absent from Streptococcus (block 3) and Buchnera (block 7) and entirely from Borrelia (block 10) (Fig. 6): in the case of *Streptococcus* spp., the first enzyme of the pathway, citrate synthase is partially present (as citZ, homologue of gltA in *E. coli *) [41], while aconitase and other key TCA cycle multi-subunit enzymes such as succinate dehydrogenase or fumarase are missing, as seen from homology searches (Data S2). These patterns of presence/absence are consistent with the general, early findings that the TCA cycle exhibits a fragmented distribution across major phylogenetic clades [42], including Borrelia [43].

Thus, in two separate cases of complex pathway evolution, using the Leucine biosynthesis (Fig. 5) and the TCA cycle (Fig. 6) as examples, it is clear that the fuzzy phylogenetic profile formalism of PathTrace allows us to capture the presence or absence of the enzyme/protein group across the target pathway with finer resolution than the RSAT approach.

In all, the homology matrix in both cases provides evidence of presence of specific enzymes in certain species/strain groups that are consistently detected in both approaches, namely PathTrace and RSAT, used as internal controls in this validation step. Again, these patterns are consistently observed for different choice of other target pathways and species collections.

### Case study: interpreting presence/absence patterns across a phylogeny

**Fig. 7.**
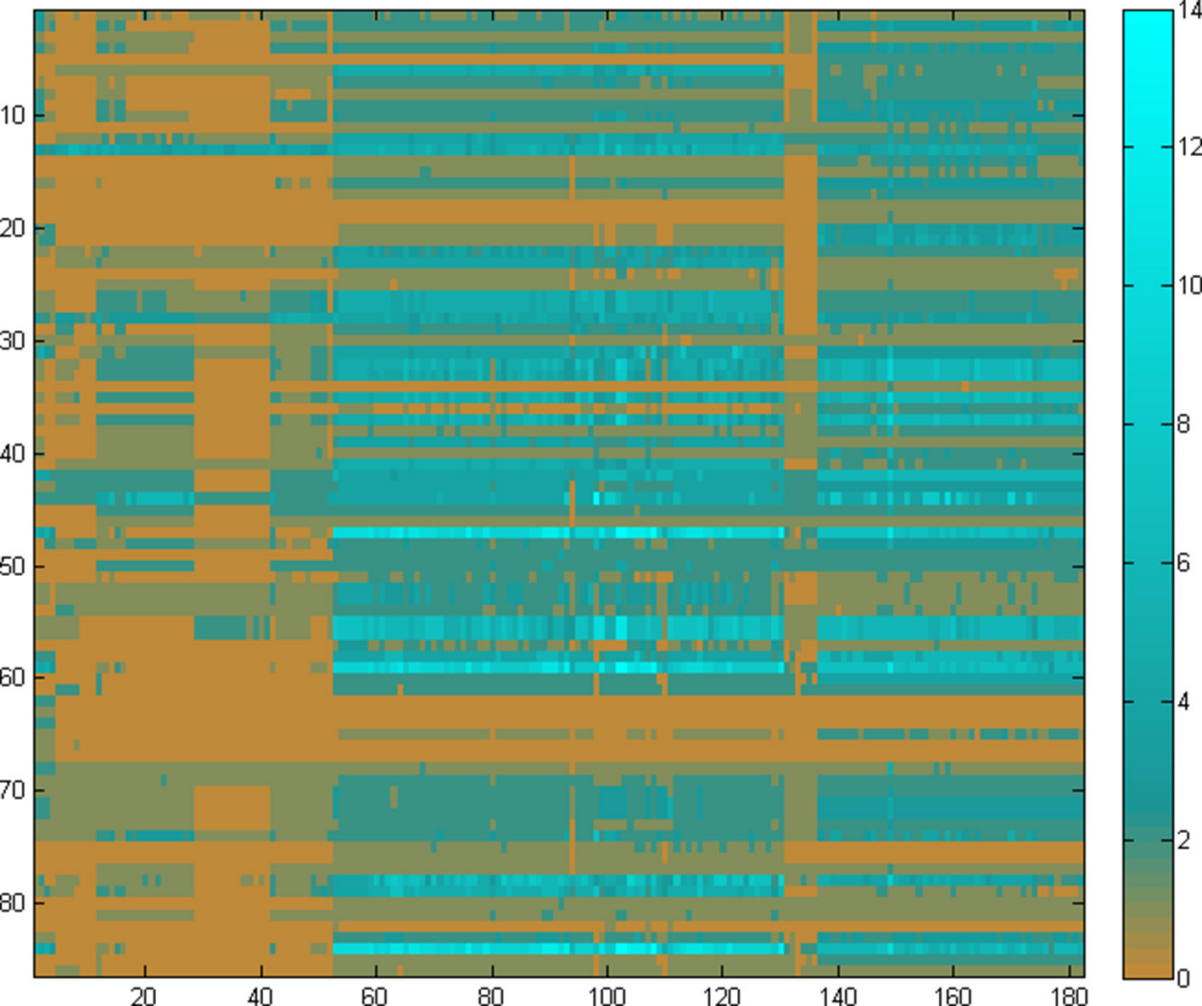
Representation of the entire homology matrix produced for the PathTrace case study. The 86 rows correspond to the enzymes that participate in the nine selected pathways, whereas the 182 columns correspond to the target genome sequences used, constructing an overall matrix of 15 652 cells. Scale from 0 to 14 (number of homologues, arbitrary value) is shown on the right.

In order to further examine PathTrace in a real-world situation and a high-throughput mode, we have applied the entire process to a set of nine metabolic pathways across a larger collection of bacterial genomes. Pathway queries were selected from common metabolic routes with a sufficient number of enzymes, 86 in total, typically well-characterized (Table 2). The target genome collection consists of ten genomes (strains) and five (most sampled) pangenomes (genera), comprising 182 single (unique) entries, thus covering a reasonable range of represented species at both the genome and pangenome levels (Table 3), following the reasoning of the findings reported above. The individual strain genomes were selected so that maximum phylogenetic variation can be captured, i.e. those most distant to the rest of the pangenome. To increase the necessary contrast, outgroups both in terms of query and target were also selected: the Glycolysis metabolic pathway Variant V (specific to Archaea) as a pathway query, and the Pyrococcus genomes (two genomes and the pangenome) as a species target set. In total, the homology matrix in this case study is composed of 86 enzymes×182 genomes (Fig. 7).

For each reference pathway, the participating protein products were selected, according to the BioCyc database, and used as queries for similarity searches. Overall, three reference genomes were used as source organisms for the corresponding pathways, namely *Escherichia coli* K-12 substr. MG1655, *Bacillus subtilis* subsp. subtilis 168 and *Pyrococcus horikoshii* OT3 (Table 2). As the presence of these pathways in the source organisms is indisputable, they have served as internal, positive controls for the development of PathTrace and the assessment of results.

**Fig. 8.**
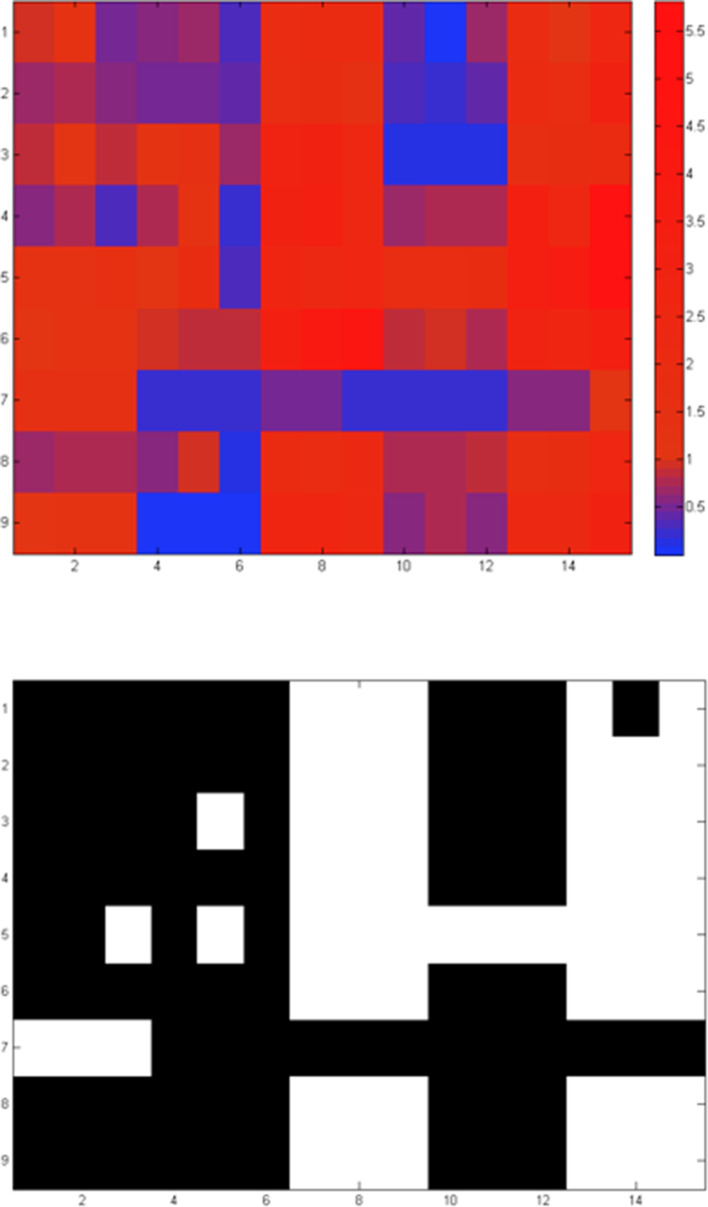
A visual representation of the fuzzy (a, top panel) and discrete (b, bottom panel) profiles of the nine pathways (rows, *y*-axis) listed in Table 2, across the 15 target genomes (columns, *x*-axis) listed in Table 3, produced by the equations in Step 3 of the PathTrace algorithm (see Methods). The colour scheme used in the fuzzy (real value) form of the profiles ranges from absence (blue) to presence (red), with intermediate values indicated partial states of the pathway. Through the application of parameter *α* (*α*=1.37), the binary profile provides a sharp overview of pathway absence or presence in the selected genomes, in a controlled manner.

Deploying the PathTrace approach, the composite pathway profiles of the nine pathways were constructed (Fig. 8), on the basis of sequence similarities (see Methods). Each profile is thus a 15-element vector (as in Table 3), with a real number value for each of the target genomes (Fig. 8a). The real-valued vectors are consequently transformed into binary vectors (Fig. 8b) through the application of a threshold for the *α* parameter (*α*=1.37 in this case, average over all genomes).

**Fig. 9.**
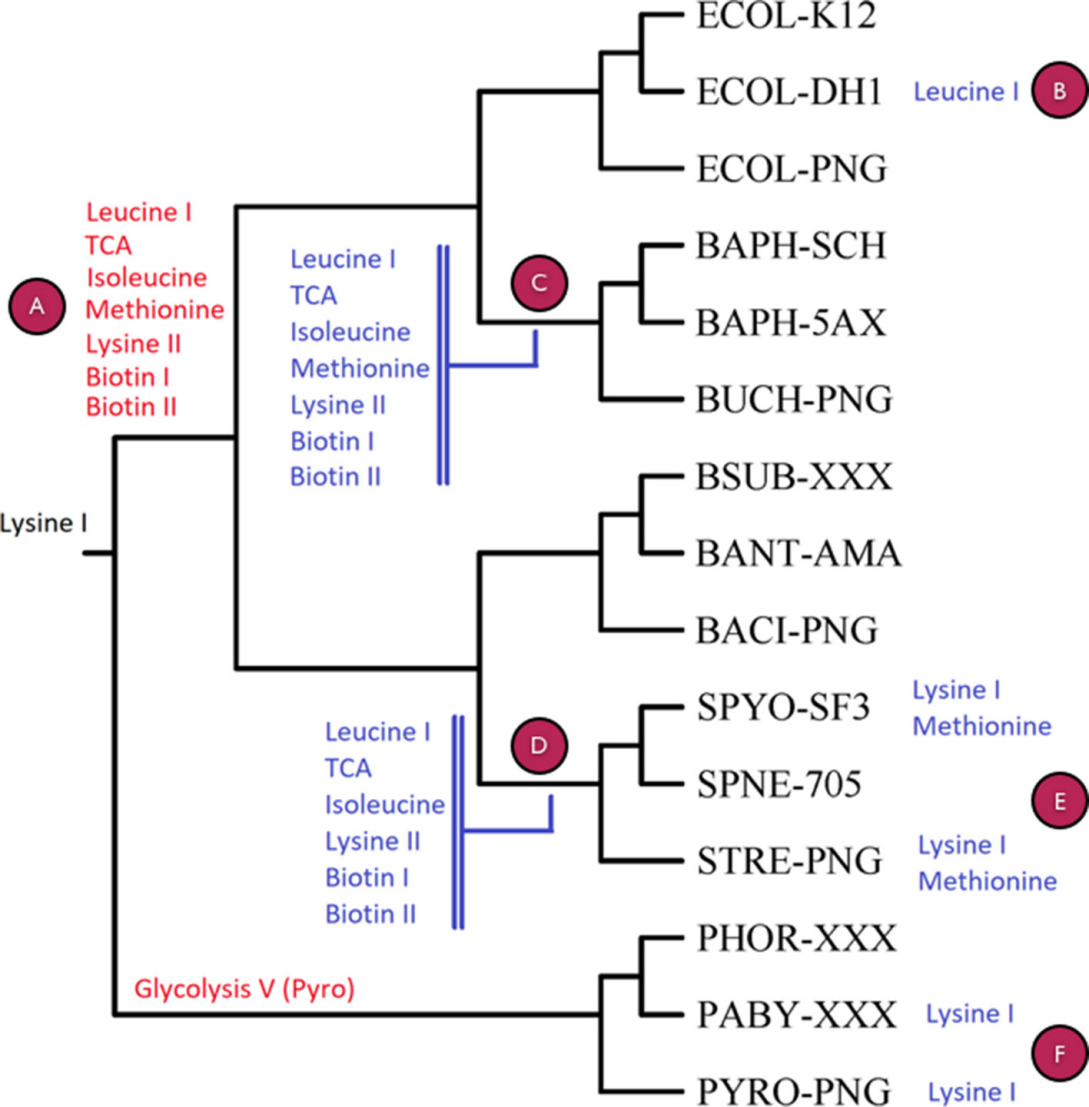
Visual representation of the PathTrace output for the nine pathways listed in Table 2, across the taxonomic tree constructed by the ten selected genomes and including the five corresponding pangenomes (encoded as: ECOL-PNG, BUCH-PNG, BACI-PNG, STRE-PNG and PYRO-PNG, as in Table 3). In each case, the pangenome is included as a ‘virtual’ genome, which includes all genomes of the species, connected to the rest of the strains at the root of the corresponding subtree. The nine pathways are shown at the root node of the subtree using the following colour scheme for parsimony-based ancestral inference: red signifies the gain (genesis) of a pathway in the corresponding subtree, blue signifies the loss of a pathway in the subtree and black denotes presence of a pathway, only evident at the root of the entire tree (in this case ‘Lysine I’). Purple circles correspond to paragraphs in section (iii) of Results, to facilitate interpretation. To assist with interpretation, more details can be found in Table S2.

To further evaluate the pathway ancestry inference, we utilize the discretized pathway profiles to assess performance in this test case (as in Table 3) coupled to biological interpretation. There are six cases that need to be discussed, which are labelled from A to F (Fig. 9). Of the nine query pathways, only Lysine biosynthesis I (DAP pathway) (no. 5 in Table 2) appears at the root of the tree, and therefore considered as universal (Fig. 9). The Glycolysis V (archaeal type) pathway (no. 7 in Table 2) is found at the root of the outgroup (* Pyrococcus* spp. – only enolase and pyruvate kinase have similarity to their *E. coli* homologues with >40 % sequence identity) while the remaining pathways appear at the root of the bacterial clade of the test set, implying that they are not detected as intact pathways in the Archaea (Fig. 9). It should be noted that this analysis represents a validation step for the method with a real-world scenario: while we have been able to interpret most of the results in a biologically meaningful manner and corroborating evidence from the literature, our intention has not been to make ‘real’ biological discoveries in this section.

Case A: the pathways for TCA cycle, Leucine I/Isoleucine/Methionine/Lysine II/Biotin I and II biosynthesis (Table 2) are found at the root of the bacterial branch, not fully detected in the archaeal outgroup (Fig. 9). The TCA cycle enzymes (no. 2 in Table 2) without homologues in the Pyrococcus clade include multiple cases, namely SucA/SucB, subunits of the E1(0) component of 2-oxoglutarate dehydrogenase, lpd – lipoamide dehydrogenase, SucD/SucC – succinyl-CoA synthetase, succinate dehydrogenase SdhB (Fe-S protein), SdhC/SdhD (membrane proteins), Fumarase (FumC), Mdh and Mqo (not shown, 11 out of 18 instances), consistent with early observations [42]. To explain the actual presence of some TCA cycle enzymes, it has been postulated that these proteins in hyperthermophilic Archaea are involved in amino acid biosynthesis [44], adapted to the environmental conditions in which these organisms thrive [45]. Isoleucine biosynthesis enzymes (no. 4 in Table 2) lack orthologues of IlvA, IlvM, IlvN, IlvH and IlvE in Pyrococcus (IlvM and possibly IlvN are absent from Archaea altogether)– 5 out of the 11 instances. Alternative isoleucine biosynthesis pathways have been detected in Bacteria, e.g [46] – not excluding this possibility for Archaea as well. Methionine biosynthesis (no. 3 in Table 2) as exhibited in Bacteria also appears to be absent in the Pyrococcus clade (e.g. no homologues of MetA or MetH can be found, two out of six instances), as the archaeal pathway has not been fully characterized [13], despite the presence of homologues in other archaeal branches (not shown). For Lysine biosynthesis II (a DAP pathway variant), homologues of Asd – semialdehyde dehydrogenase, DapB, DapD, DapF and LysA, i.e. five out of nine instances, are missing from Pyrococcus, clearly indicating the absence of this pathway variant (no. 8 in Table 2), as opposed to the AAA pathway for lysine biosynthesis in Archaea [47], potentially connected to arginine [48] or leucine [49] biosynthesis. Finally, biotin biosynthesis pathways (no. 6 and no. 9 in Table 2) appear to be absent from Pyrococcus, as a representative for the archaeal domain: for biotin I, only FabG, BioF, BioA (3 out of 11 instances) have highly similar genes in this genus (while most genes are present in some Archaea, with the possible exception of BioB, BioD, BioH, FabB, FabH and FabI – not shown); for biotin II, BioF and BioK homologues are present in Pyrococcus (two out of five instances); this pathway appears to be present in Archaea in general, justifying the presence of BirA [50] and of course the biotin regulon [51].

Case B: The loss of Leucine biosynthesis I in *E. coli* DH10b (Fig. 9) has been commented above (Fig. 5), an interesting case of detection of pathway absence with biochemical and genetic evidence to support it.

Case C: The loss of Leucine biosynthesis I in *Buchnera* spp. is evident, with TyrB and IlvE being absent. The TCA cycle is also incomplete, with key enzymes missing, for example succinate dehydrogenase or fumarase subunit homologues, amongst others [43]; only homologues for Icd, SucA, SucB and Lpd are detectable, i.e. 4 out of 18 instances [52]. Interestingly, SucA, SucB and Lpd are involved in the 2-oxoglutarate decarboxylation to succinyl-CoA (see also, relevant biocyc entry), potentially justifying the presence of only those homologues (3 out of 18 instances). For Isoleucine biosynthesis, half of the enzymes are detected (6 out of 11 instances), namely IlvG, IlvI, IlvH (all subunits of acetolactate synthase), IlvB (involved in ILV biosynthesis), IlvC and IlvD – their role in this species is somewhat unclear, as the last enzyme in this pathway (IlvE) is not detectable; its function might be complemented by another aminotransferase. Buchnera lacks enzyme homologues for methionine biosynthesis except MetE [53], pointing to the possibility of production of methionine from cystathionine (see also, relevant biocyc entry); we reason that this pathway has been lost in the entire pangenome of this clade. The Lysine II variant has been lost in this branch, as SpoV genes as well as DapL are not detectable. It should be noted that Buchnera synthesizes both leucine and lysine, through other pathway variants not used in this study [54]. For Biotin I biosynthesis, BioC/FabH/BioF are missing (3 out of 11 instances), while for Biotin II biosynthesis, BioW/BioF are not detectable across the pangenome. These results for Buchnera are consistent with its nature as an extreme case of genome reduction [55], and clearly point to gene loss as a major factor shaping both gene content and metabolic capabilities [5] (Fig. 9).

Case D: A similar group of undetectable pathways emerges for the *Streptococcus clade*, with the exception of Methionine biosynthesis, which presents a more complex pattern (Fig. 9). Indeed, it is established that many amino acid biosynthesis pathways might be absent from Streptococcus pyogenes (including Leucine, Methionine, Isoleucine), fewer in the case of *S. pneumoniae* [56]. For Leucine I biosynthesis, TyrB is not detectable at the pangenome level (except *S. pneumoniae* and *S. equinus *), while all enzymes except IlvE (branched-chain aminotransferase) are missing from *S. pyogenes *. The *Streptococcus pangenome* apparently lacks the TCA cycle (at least components of succinate dehydrogenase, e.g. SucD, SdhB exhibit a limited distribution) as well as the Isoleucine (IlvM/IlvN missing) and Biotin I (BioC/BioH missing)/II (limited distribution of certain enzyme homologues across the pangenome) biosynthesis pathways [57]. For Lysine II, DapG/LysC as queries have similarities to potentially uncharacterized aspartokinases in *S. pneumoniae *, while SpoV genes and DapF are missing (except in *S. pneumoniae *); all genes with the exception of a DapA homologue are missing from *S. pyogenes *, reflecting a genuine, established biological fact [58–60] (Fig. 9). In this complex case, PathTrace returns its results according to the generic threshold imposed, whereas in the Buchnera case the number of homologues is relatively restricted, in the case of *Streptococcus* it is evident that in certain cases the presence of a pathway is more difficult to establish, as there is a much wider variation in the number of enzyme homologues across strains and species.

Case E: The particular losses of Lysine I (no. 5 in Table 2) and Methionine (no. 3 in Table 2) biosynthesis detected by PathTrace for *S. pyogenes* SF370 and the *Streptococcus pangenome* are consistent with the absence of most enzymes in the former strain and their fragmented distribution across other strains [59]. For instance, DapF (lysine biosynthesis) is present only in *S. pneumoniae* and *S. agalactiae* (Table S2). In the case of *S. pneumoniae *, while most enzymes have homologues in the case of Lysine I biosynthesis, Asd, ArgD, DapE and DapF (four out of nine instances) are missing; this might be due to the threshold imposed by the algorithm (Table S2). Similarly, *S. pneumoniae* lacks MetE in Methionine biosynthesis (one out of six instances), while *S. pyogenes* only possesses MetB and MetC enzymes. For the entire pangenome, a fragmented distribution of homologues is observed, with MetH for instance being partially absent (Fig. 9). Overall, this case signifies the challenge of exact pathway detection in complex pangenomes and the need for more accurate, taxon-specific values for the threshold of detection.

Case F: Finally, in the case of the *Pyrococcus clade*, Lysine I biosynthesis (no. 5 in Table 2) is detected as present in *P. horikoshii* (with DapA/ArgD/DapE present), but potentially absent from *P. abyssi* and the pangenome – where it is known to be replaced by the AAA lysine biosynthesis pathway [61]. The presence of the above homologues is not fully understood, to our knowledge – these proteins might be involved in processes other than lysine biosynthesis.

All the above results are available on the Figshare repository (see, Data Summary) and might be reproduced either in automated or interactive fashion, using as queries the sequences from the nine pathways documented in FASTA format in Data S1 and targets the specific databases restricted to the taxon (strain, species, genus or pangenome) of interest.

The detailed case study results for the assessment of PathTrace are consistent with our knowledge of pathway evolution, supported by corroborating evidence from the literature. It also demonstrates the difficulties of establishing pathway presence or absence, in particular with a single threshold – an ongoing challenge in fields such as similarity searches and clustering [62].

**Fig. 10.**
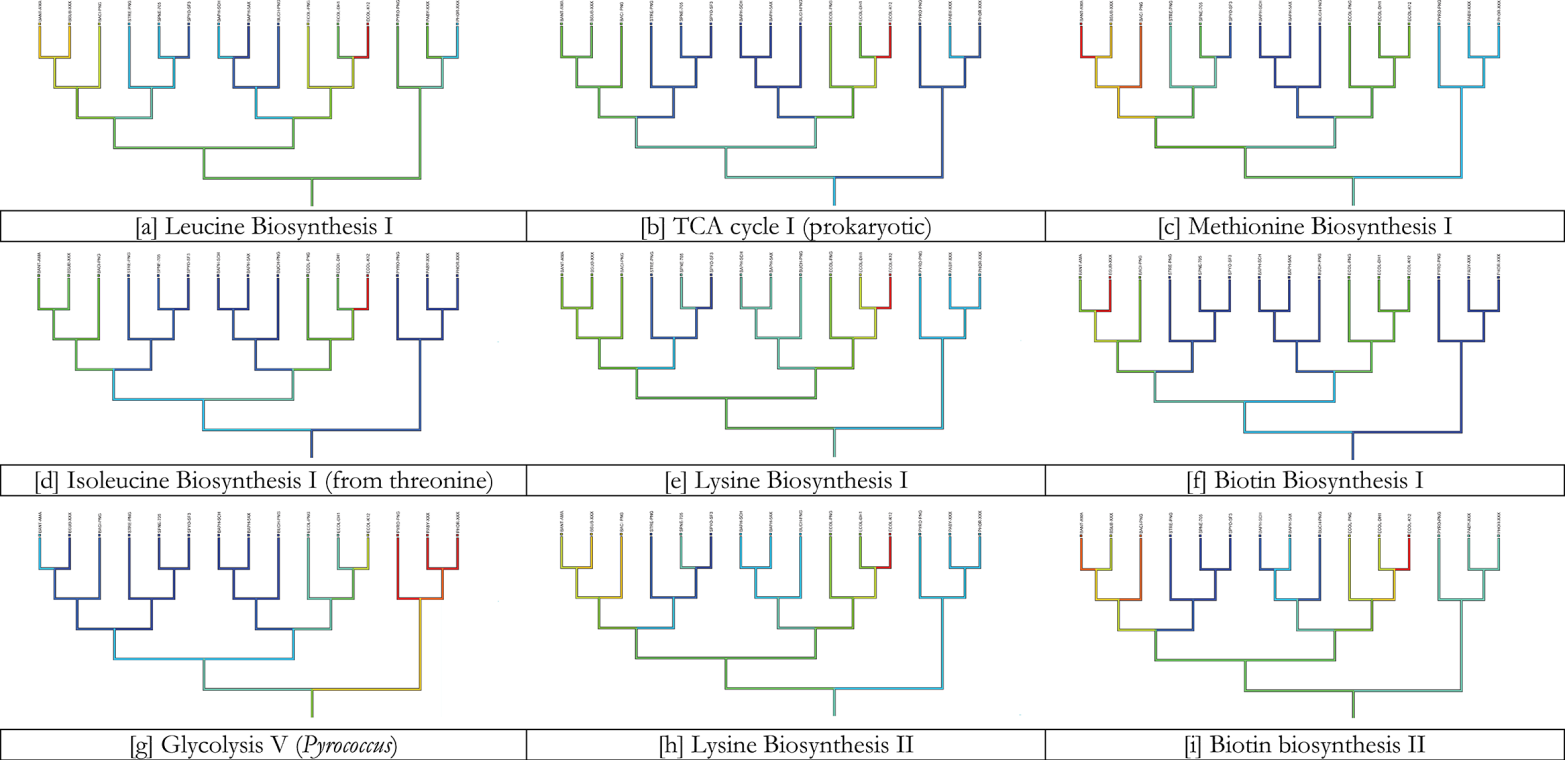
Comparison of PathTrace with the Mesquite suite using the selected nine pathways listed in Table 2. The colour scheme used by Mesquite ranges from red (denoting presence) to blue (denoting absence), with intermediate colours defining partial status (see also Fig. S1). The taxonomic tree utilized here is identical to the tree used in the PathTrace use case (Fig. 9), with a 90 degree rotation. Inspection of the nine panels reveals that both Mesquite and PathTrace establish similar evolutionary scenarios of the query pathways.

To further compare the last element of PathTrace, namely the ancestral inference given a pathway profile, we deployed Mesquite, a suite for evolutionary inference [35]. Mesquite is not capable to execute the early phases of the PathTrace protocol, but can be used for a parsimony-based inference of ancestral states. To this end, we used the fuzzy, real-value profiles of the nine selected pathways, and applied the maximum parsimony process of Mesquite using default parameters. The produced patterns closely follow the results of PathTrace as can be seen in Fig. 10 – and in more detail in Fig. S1 for individual pathways and raw output, signifying that parsimony-based evolutionary histories can be reproduced consistently.

This final, complex section summarizes the presence/absence patterns of query pathways across the target genomes and their corresponding clades and demonstrates that the PathTrace pathway profiles capture succinctly the distribution of these patterns with reasonable accuracy, reflecting genuine biological knowledge supported by existing knowledge. While it has been difficult to interpret partial presence of pathways (i.e. a fraction of their enzyme sequences), the most challenging issue has been the assertion of absence for certain reactions/pathways, as negative results can rarely be established, or in fact reported in the literature.

## Discussion

By extending the notion of a phylogenetic profile to a composite group represented by the corresponding enzyme profiles of a pathway against a target selection of genomes, and typically organized into a pangenome, PathTrace is able to detect unambiguously the presence or absence of a pathway across a phylogeny. While the algorithm is based on parsimony and inspired by previous, similar implementations of phylogenetic profiling, it is evident that its performance is significantly increased when coherent, phylogenetically similar genome collections are used as targets, eliminating individual effects deriving from gene gain or loss. Indeed, following extensive experimentation, we have established the advantage of pangenome collections for a more accurate detection of pathway groups. It is evident that query pathways, represented as groups of genes, are less sensitive to parameters of parsimony-based algorithms for gain or loss [22]; however, the crisp decision for presence or absence remains a bottleneck.

More sophisticated models using taxon-specific values and genome-size normalization [63] or dynamical models of gene acquisition and loss impacting pathways [64] are directions for future algorithm development. Issues that also need further investigation include the effect of multiple paralogues on parameter choice (thresholds, e.g. [65]), the differential treatment of enzymes with respect to genome structure or pathway topology [66], the mode and tempo of horizontal gene transfers [67], the nature of ecological characteristics of target organisms with respect to speciation and innovation [68], as well as integration of PathTrace with related tools into software pipelines and relevant resources [69]. Those developments, coupled with the ever-growing availability of pangenome ensembles will facilitate the inference of ancestral states for entire pathways, helping us decipher the emergence and loss of entire functional modules within a broader phylogenetic and ecological context.

## Supplementary Data

Supplementary material 1
