# Supplementary material for: Ancestral state reconstruction of metabolic pathways across pangenome ensembles
Source: Microb Genom. 2020 Sep 14;6(11):mgen000429. doi: 10.1099/mgen.0.000429 (PMC7725326; doi:10.1099/mgen.0.000429)
Supplement: Supplementary material 1 [file mgen-6-429-s001.pdf]

Ancestral state reconstruction of metabolic pathways across pangenome ensembles

Fotis E. Psomopoulos[1], Jacques van Helden[2] , Claudine Médigue[3], Anastasia Chasapi[4] and Christos A. Ouzounis[4*]

[1] Institute of Applied Biosciences (INAB), Center for Research & Technology Hellas (CERTH), GR-57001 Thessalonica, Greece

[2] Lab. Technological Advances for Genomics & Clinics (TAGC), Université d'Aix-Marseille (AMU), INSERM Unit U1090, 163, Avenue de Luminy, 13288 Marseille cedex 09, France

[3] UMR 8030, CNRS, Université Evry-Val-d'Essonne, CEA, Institut de Biologie François Jacob – Genoscope, Laboratoire d'Analyses Bioinformatiques pour la Génomique et le Métabolisme, Evry, France

[4] Biological Computation & Process Laboratory (BCPL), Chemical Process & Energy Resources Institute (CPERI), Center for Research & Technology Hellas (CERTH), GR-57001 Thessalonica, Greece

Microbial Genomics – version 1.021

Supporting Information

There are overall 249 columns – (x-axis in **Figures 4-6**), corresponding to an equal number of genomes across 10 different pangenomes, listed in the following order: columns 1-4: *Pyrococcus* collection, cols 5-24: *Mycobacterium* collection, cols 25-72: *Streptococcus* collection, cols 73-150: *Bacillus* collection, cols 151-177: *Staphylococcus* collection, cols 178-181: *Wolbachia* collection, cols 182-187: *Buchnera* collection, cols 188-233: *Escherichia / Shigella* collection, cols 234-241: *Neisseria* collection, cols 242-249: *Borrelia* collection.

Table S1: Bacterial pangenome data collections

Data retrieved from: bacteria.ensembl.org, (rel. 16: 10 collections / 249 genomes / 934,772 sequences).

Pyrococcus collection (columns 1-4) - 8,225 sequences

number	species/strain name	internal code
1	*Pyrococcus abyssi*	PABY-XXX
2	*Pyrococcus furiosus*	PFUR-XXX
3	*Pyrococcus horikoshii*	PHOR-XXX
4	*Pyrococcus kodakaraensis*	PKOD-XXX

Mycobacterium collection (columns 5-24) - 91,032 sequences

number	species/strain name	internal code
1	*Mycobacterium abscessus*	MABS-XXX
2	*Mycobacterium avium*	MAVI-XXX
3	*Mycobacterium bovis* af2122 97	MBOV-AF2
4	*Mycobacterium bovis* pasteur 1173p2	MBOV-PAS
5	*Mycobacterium bovis* tokyo 172	MBOV-TOK
6	*Mycobacterium gilvum*	MGIL-XXX
7	*Mycobacterium leprae* br4923	MLEP-BR4
8	*Mycobacterium leprae* tn	MLEP-TNX
9	*Mycobacterium marinum*	MMAR-XXX
10	*Mycobacterium paratuberculosis*	MPAR-XXX
11	*Mycobacterium smegmatis*	MSME-XXX
12	*Mycobacterium tuberculosis* atcc 25177	MTUB-ATC
13	*Mycobacterium tuberculosis* cdc1551	MTUB-CDC
14	*Mycobacterium tuberculosis* h37rv	MTUB-H37
15	*Mycobacterium tuberculosis* kzn 1435	MTUB-KZN
16	*Mycobacterium ulcerans*	MULC-XXX
17	*Mycobacterium vanbaalenii*	MVAN-XXX
18	*Mycobacterium* sp. jls	MYCO-JLS
19	*Mycobacterium* sp. kms	MYCO-KMS
20	*Mycobacterium* sp. mcs	MYCO-MCS

Streptococcus collection (columns 25-72) - 94,513 sequences

number	species/strain name	internal code
1	*Streptococcus agalactiae* ia	SAGA-IAX
2	*Streptococcus agalactiae* iii	SAGA-III

3	*Streptococcus agalactiae* v	SAGA-VXX
4	*Streptococcus* dysgalactiae	SDYS-XXX
5	*Streptococcus equi*	SEQU-XXX
6	*Streptococcus equi* mgcs10565	SEQU-MGC
7	*Streptococcus equi* zooepidemicus	SEQU-ZOO
8	*Streptococcus gallolyticus*	SGAL-XXX
9	*Streptococcus gordonii*	SGOR-XXX
10	*Streptococcus mitis*	SMIT-XXX
11	*Streptococcus mutans* atcc 700610	SMUT-ATC
12	*Streptococcus mutans* nn2025	SMUT-NN2
13	*Streptococcus pneumoniae* 70585	SPNE-705
14	*Streptococcus pneumoniae* a19	SPNE-A19
15	*Streptococcus pneumoniae* atcc 700669	SPNE-AT7
16	*Streptococcus pneumoniae* atcc baa 255	SPNE-ATB
17	*Streptococcus pneumoniae* cgsp14	SPNE-CGS
18	*Streptococcus pneumoniae* d39	SPNE-D39
19	*Streptococcus pneumoniae* g54	SPNE-G54
20	*Streptococcus pneumoniae* hungary19a 6	SPNE-HUN
21	*Streptococcus pneumoniae* jja	SPNE-JJA
22	*Streptococcus pneumoniae* p1031	SPNE-P10
23	*Streptococcus pneumoniae* taiwan19f 14	SPNE-TAI
24	*Streptococcus pneumoniae* tigr4	SPNE-TIG
25	*Streptococcus pyogenes* atcc baa 595	SPYO-ATC
26	*Streptococcus pyogenes* m18	SPYO-M18
27	*Streptococcus pyogenes* m28	SPYO-M28
28	*Streptococcus pyogenes* m2	SPYO-M2X
29	*Streptococcus pyogenes* m49	SPYO-M49
30	*Streptococcus pyogenes* m4	SPYO-M4X
31	*Streptococcus pyogenes* m5	SPYO-M5X
32	*Streptococcus pyogenes* m6	SPYO-M6X
33	*Streptococcus pyogenes* mgas2096	SPYO-MG6
34	*Streptococcus pyogenes* mgas5005	SPYO-MG5
35	*Streptococcus pyogenes* mgas9429	SPYO-MG9
36	*Streptococcus pyogenes* sf370	SPYO-SF3
37	*Streptococcus pyogenes* ssi 1	SPYO-SSI
38	*Streptococcus sanguinis*	SSAN-XXX
39	*Streptococcus suis* 05zyh33	SSUI-05Z
40	*Streptococcus suis* 98hah33	SSUI-98H

41	*Streptococcus suis* bm407	SSUI-BM4
42	*Streptococcus suis* gz1	SSUI-GZ1
43	*Streptococcus suis* p1 7	SSUI-P17
44	*Streptococcus suis* sc84	SSUI-SC8
45	*Streptococcus thermophilus* atcc baa 250	STHE-AT2
46	*Streptococcus thermophilus* atcc baa 491	STHE-AT4
47	*Streptococcus thermophilus* cnrz 1066	STHE-CNR
48	*Streptococcus uberis*	SUBE-XXX

Bacillus collection (columns 73-150) - 418,590 sequences

number	species/strain name	internal code
1	*Bacillus amyloliquefaciens*	BAMY-XXX
2	*Bacillus anthracis* a0248	BANT-A02
3	*Bacillus anthracis* ames ancestor	BANT-AMA
4	*Bacillus anthracis* ames	BANT-AME
5	*Bacillus anthracis* cdc 684	BANT-CDC
6	*Bacillus anthracis* sterne	BANT-STE
7	*Bacillus cereus* 03bb102	BCER-03B
8	*Bacillus cereus* 172560w	BCER-172
9	*Bacillus cereus* 95 8201	BCER-958
10	*Bacillus cereus* ah1271	BCER-H71
11	*Bacillus cereus* ah1272	BCER-H72
12	*Bacillus cereus* ah1273	BCER-H73
13	*Bacillus cereus* ah187	BCER-H18
14	*Bacillus cereus* ah603	BCER-H60
15	*Bacillus cereus* ah621	BCER-H62
16	*Bacillus cereus* ah676	BCER-H67
17	*Bacillus cereus* ah820	BCER-H82
18	*Bacillus cereus* atcc 10876	BCER-A76
19	*Bacillus cereus* atcc 10987	BCER-A87
20	*Bacillus cereus* atcc 14579	BCER-A79
21	*Bacillus cereus* atcc 4342	BCER-A42
22	*Bacillus cereus* b4264	BCER-B42
23	*Bacillus cereus* bdrd bcer4	BCER-BCE
24	*Bacillus cereus* bdrd st196	BCER-ST1
25	*Bacillus cereus* bdrd st24	BCER-ST4
26	*Bacillus cereus* bdrd st26	BCER-ST6
27	*Bacillus cereus* bgsc 6e1	BCER-6E1

28	*Bacillus cereus* cytotoxis	BCER-CYT
29	*Bacillus cereus* f65185	BCER-F65
30	*Bacillus cereus* g9842	BCER-G98
31	*Bacillus cereus* m1293	BCER-M12
32	*Bacillus cereus* mm1550	BCER-MM1
33	*Bacillus cereus* mm3	BCER-MM3
34	*Bacillus cereus* q1	BCER-Q1X
35	*Bacillus cereus* r309803	BCER-R30
36	*Bacillus cereus* rock1 15	BCER-R15
37	*Bacillus cereus* rock1 3	BCER-R3X
38	*Bacillus cereus* rock3 28	BCER-R28
39	*Bacillus cereus* rock3 29	BCER-R29
40	*Bacillus cereus* rock3 42	BCER-R42
41	*Bacillus cereus* rock3 44	BCER-R44
42	*Bacillus cereus* rock4 18	BCER-R18
43	*Bacillus cereus* rock4 2	BCER-R2X
44	*Bacillus cereus* var anthracis	BCER-ANT
45	*Bacillus cereus* zk	BCER-ZKX
46	*Bacillus clausii*	BCLA-XXX
47	*Bacillus halodurans*	BHAL-XXX
48	*Bacillus licheniformis* goettingen	BLIC-GOE
49	*Bacillus licheniformis* novozymes	BLIC-NOV
50	*Bacillus megaterium* atcc 12872	BMEG-ATC
51	*Bacillus megaterium* dsm 319	BMEG-DSM
52	*Bacillus mycoides* dsm 2048	BMYC-DSM
53	*Bacillus mycoides* rock1 4	BMYC-R4X
54	*Bacillus mycoides* rock3 17	BMYC-R17
55	*Bacillus pseudofirmus*	BFIR-PSE
56	*Bacillus pseudomycoides*	BMYC-PSE
57	*Bacillus pumilus*	BPUM-XXX
58	*Bacillus selenitireducens*	BSEL-XXX
59	*Bacillus subtilis*	BSUB-XXX
60	*Bacillus thuringiensis* atcc 10792	BTHU-ATC
61	*Bacillus thuringiensis* bgsc 4aj1	BTHU-4AJ
62	*Bacillus thuringiensis* bgsc 4aw1 andalous	BTHU-4AW
63	*Bacillus thuringiensis* bgsc 4ba1 pondicheri	BTHU-4BA
64	*Bacillus thuringiensis* bgsc 4bd1 huazhong	BTHU-4BD
65	*Bacillus thuringiensis* bgsc 4cc1 pulsiensis	BTHU-4CC

66	*Bacillus thuringiensis* bgsc 4y1 tochigiensis	BTHU-4YI
67	*Bacillus thuringiensis* bmb171	BTHU-BMB
68	*Bacillus thuringiensis* bt407	BTHU-BT4
69	*Bacillus thuringiensis*	BTHU-XXX
70	*Bacillus thuringiensis* ibl200	BTHU-IB2
71	*Bacillus thuringiensis* ibl4222	BTHU-IB4
72	*Bacillus thuringiensis* konkukian	BTHU-KON
73	*Bacillus thuringiensis* t01001	BTHU-T01
74	*Bacillus thuringiensis* t03a001 kurstaki	BTHU-T03
75	*Bacillus thuringiensis* t04001 sotto	BTHU-T04
76	*Bacillus thuringiensis* t13001 pakistani	BTHU-T13
77	*Bacillus tusciae*	BTUS-XXX
78	*Bacillus weihenstephanensis*	BWEI-XXX

Staphylococcus collection (columns 151-177) - 71,304 sequences

number	species/strain name	internal code
1	*Staphylococcus aureus* 04 02981	SAUR-04X
2	*Staphylococcus aureus* bovine rf122	SAUR-BOV
3	*Staphylococcus aureus* col	SAUR-COL
4	*Staphylococcus aureus* ed133	SAUR-ED1
5	*Staphylococcus aureus* ed98	SAUR-ED9
6	*Staphylococcus aureus* jh1	SAUR-JH1
7	*Staphylococcus aureus* jh9	SAUR-JH9
8	*Staphylococcus aureus* jkd6008	SAUR-J60
9	*Staphylococcus aureus* jkd6159	SAUR-J61
10	*Staphylococcus aureus* mrsa252	SAUR-MRS
11	*Staphylococcus aureus* mssa476	SAUR-MSS
12	*Staphylococcus aureus* mu3	SAUR-MU3
13	*Staphylococcus aureus* mu50	SAUR-MU5
14	*Staphylococcus aureus* mw2	SAUR-MW2
15	*Staphylococcus aureus* n315	SAUR-N31
16	*Staphylococcus aureus* nctc 8325	SAUR-NCT
17	*Staphylococcus aureus* newman	SAUR-NEW
18	*Staphylococcus aureus* st398	SAUR-ST3
19	*Staphylococcus aureus* tch1516	SAUR-TCH
20	*Staphylococcus aureus* tw20	SAUR-TW2
21	*Staphylococcus aureus* usa300	SAUR-USA
22	*Staphylococcus carnosus*	SCAR-XXX

23	*Staphylococcus epidermidis* atcc 12228	SEPI-AT1
24	*Staphylococcus epidermidis* atcc 35984	SAPI-AT3
25	*Staphylococcus haemolyticus*	SHAE-XXX
26	*Staphylococcus lugdunensis*	SLUG-XXX
27	*Staphylococcus saprophyticus*	SSAP-XXX

Wolbachia collection (columns 178-181) - 4,425 sequences

number	species/strain name	internal code
1	*Wolbachia* sp brugia malayi	WOLB-BRU
2	*Wolbachia* sp drosophila simulans	WOLB-DRO
3	*Wolbachia pipientis* culex pipiens	WPIP-CUL
4	*Wolbachia pipientis* wmel	WPIP-WME

Buchnera collection (columns 182-187) - 3,106 sequences

number	species/strain name	internal code
1	*Buchnera aphidicola* 5a	BAPH-5AX
2	*Buchnera aphidicola* baizongia	BAPH-BAI
3	*Buchnera aphidicola* cinara	BAPH-CIN
4	*Buchnera aphidicola* schizaphis	BAPH-SCH
5	*Buchnera aphidicola* tokyo 1998	BAPH-TOK
6	*Buchnera aphidicola* tuc7	BAPH-TUC

Escherichia / *Shigella* collection (columns 188-233) - 217,904 sequences

number	species/strain name	internal code
1	*Escherichia coli* 04	ECOL-042
2	*Escherichia coli* 55989	ECOL-559
3	*Escherichia coli* atcc 27325	ECOL-A27
4	*Escherichia coli* atcc 33849	ECOL-A33
5	*Escherichia coli* atcc 8739	ECOL-A87
6	*Escherichia coli* bl21 de3 jgi	ECOL-JGI
7	*Escherichia coli* bl21 de3 kribb	ECOL-KRI
8	*Escherichia coli* bl21	ECOL-BL2
9	*Escherichia coli* bw2952	ECOL-BW2
10	*Escherichia coli* dh10b	ECOL-DH1
11	*Escherichia coli* ec4115	ECOL-EC4
12	*Escherichia coli* edl933	ECOL-EDL
13	*Escherichia coli* k12	ECOL-K12
14	*Escherichia coli* o103 h2	ECOL-103
15	*Escherichia coli* o111 h	ECOL-111

16	*Escherichia coli* o127 h6	ECOL-127
17	*Escherichia coli* o139 h28	ECOL-139
18	*Escherichia coli* o157 h7 tw14588	ECOL-157
19	*Escherichia coli* o17 k52 h18	ECOL-017
20	*Escherichia coli* o18 k1 h7	ECOL-018
21	*Escherichia coli* o1 k1 apec	ECOL-001
22	*Escherichia coli* o26 h11	ECOL-026
23	*Escherichia coli* o45 k1	ECOL-045
24	*Escherichia coli* o55 h7	ECOL-055
25	*Escherichia coli* o6	ECOL-006
26	*Escherichia coli* o6 k15 h31	ECOL-K15
27	*Escherichia coli* o7 k1	ECOL-007
28	*Escherichia coli* o81	ECOL-081
29	*Escherichia coli* o8	ECOL-008
30	*Escherichia coli* o9 h4	ECOL-009
31	*Escherichia coli* rel606	ECOL-REL
32	*Escherichia coli* sakai	ECOL-SAK
33	*Escherichia coli* se11	ECOL-SE1
34	*Escherichia coli* se15	ECOL-SE5
35	*Escherichia coli* sms 3 5	ECOL-SMS
36	*Escherichia coli* tw14359	ECOL-TW1
37	*Escherichia coli* uti89	ECOL-UTI
38	*Escherichia fergusonii*	EFER-XXX
39	*Shigella boydii* 18	SBOY-018
40	*Shigella boydii* 4	SBOY-004
41	*Shigella dysenteriae*	SDYS-XXX
42	*Shigella flexneri* 2457t	SFLE-245
43	*Shigella flexneri* 301	SFLE-301
44	*Shigella flexneri* 5b	SFLE-05B
45	*Shigella flexneri* x	SFLE-00X
46	*Shigella sonnei*	SSON-XXX

Neisseria collection (columns 234-241) - 16,349 sequences

number	species/strain name	internal code
1	*Neisseria gonorrhoeae* atcc 700825	NGON-ATC
2	*Neisseria gonorrhoeae* nccp11945	NGON-NCC
3	*Neisseria meningitidis* 053442	NMEN-053
4	*Neisseria meningitidis* 2a	NMEN-2AX

5	*Neisseria meningitidis* 8013	NMEN-801
6	*Neisseria meningitidis* a	NMEN-AXX
7	*Neisseria meningitidis* alpha14	NMEN-ALP
8	*Neisseria meningitidis* b	NMEN-BXX

Borrelia collection (columns 242-249) - 9,324 sequences

number	species/strain name	internal code
1	*Borrelia afzelii*	BAFZ-XXX
2	*Borrelia burgdorferi* dsm 4680	BBUR-DSM
3	*Borrelia burgdorferi* zs7	BBUR-ZS7
4	*Borrelia duttonii*	BDUT-XXX
5	*Borrelia garinii*	BGAR-XXX
6	*Borrelia hermsii*	BHER-XXX
7	*Borrelia recurrentis*	BREC-XXX
8	*Borrelia turicatae*	BTUR-XXX

Table S2: Scenarios for evolutionary gain or loss for nine query pathways

Color-coded scheme corresponding to **Figure 9** as an aid for its interpretation.

(see next page)

	PYRO–PNG	PABY–XXX	PHOR–XXX	STRE–PNG	SPNE–705	SPYO–SF3	BACI–PNG	BANT–AMA	BSUB–XXX	BUCH–PNG	BAPH–5AX	BAPH–SCH	ECOL–PNG	ECOL–DH1	ECOL–K12
lysine I	1.31	1.33	1.56	1.17	1.89	0.33	3.47	3.44	3.33	1.74	1.78	1.89	3.43	3.78	5.00
leucine I	1.00	1.33	0.50	0.62	0.67	0.33	1.92	2.00	2.00	0.44	0.00	0.67	1.91	1.17	2.50
TCA	0.69	0.78	0.56	0.52	0.50	0.39	1.76	1.83	1.50	0.33	0.22	0.39	2.10	1.78	3.00
isoleucine	0.55	0.73	0.27	0.77	1.27	0.18	3.01	3.27	2.45	0.71	0.73	0.73	3.34	2.45	5.82
methionine	0.83	1.00	0.83	1.22	1.50	0.67	2.96	3.00	2.50	0.17	0.17	0.17	1.69	1.83	2.00
lysine II	0.71	0.75	0.75	0.61	0.92	0.17	2.18	1.92	2.25	0.76	0.75	0.83	1.72	1.83	2.58
biotin I	1.02	1.09	0.91	0.96	0.82	0.82	5.18	5.00	7.09	0.82	0.91	0.73	4.34	4.18	4.55
biotin II	1.05	1.20	1.20	0.07	0.00	0.00	2.78	2.80	2.40	0.60	0.80	0.60	2.30	2.20	3.00
glycolysis V	1.38	1.38	1.38	0.26	0.25	0.25	0.46	0.50	0.25	0.25	0.25	0.25	0.60	0.63	1.13

	PYRO–PNG	PABY–XXX	PHOR–XXX	STRE–PNG	SPNE–705	SPYO–SF3	BACI–PNG	BANT–AMA	BSUB–XXX	BUCH–PNG	BAPH–5AX	BAPH–SCH	ECOL–PNG	ECOL–DH1	ECOL–K12
lysine I	0	0	1	0	1	0	1	1	1	1	1	1	1	1	1
leucine I	0	0	0	0	0	0	1	1	1	0	0	0	1	0	1
TCA	0	0	0	0	0	0	1	1	1	0	0	0	1	1	1
isoleucine	0	0	0	0	0	0	1	1	1	0	0	0	1	1	1
methionine	0	0	0	0	1	0	1	1	1	0	0	0	1	1	1
lysine II	0	0	0	0	0	0	1	1	1	0	0	0	1	1	1
biotin I	0	0	0	0	0	0	1	1	1	0	0	0	1	1	1
biotin II	0	0	0	0	0	0	1	1	1	0	0	0	1	1	1
glycolysis V	1	1	1	0	0	0	0	0	0	0	0	0	0	0	0

never present
lost in this clade
inherited vertically

Figure S1: Ancestral reconstructions for nine query pathways

Ancestral state reconstructions for the nine query pathways of `PathTrace` -- using `Mesquite`.

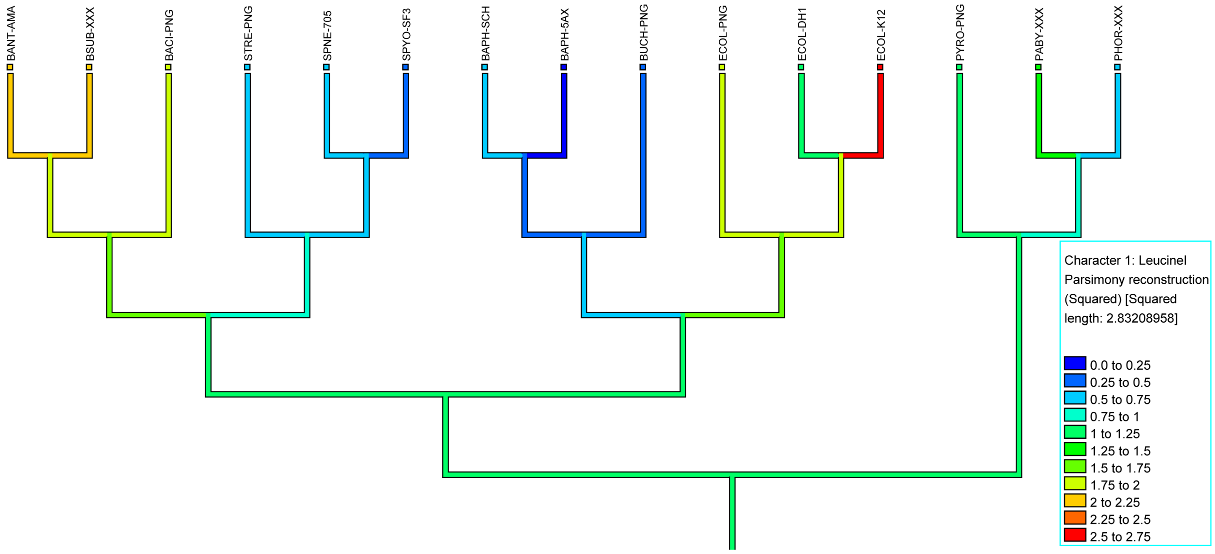

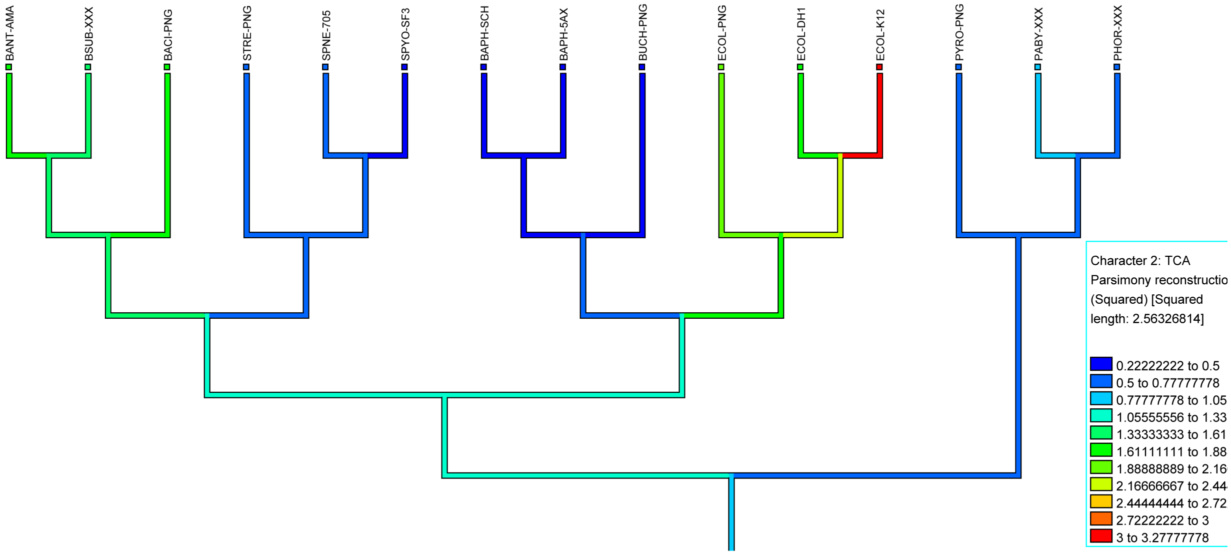

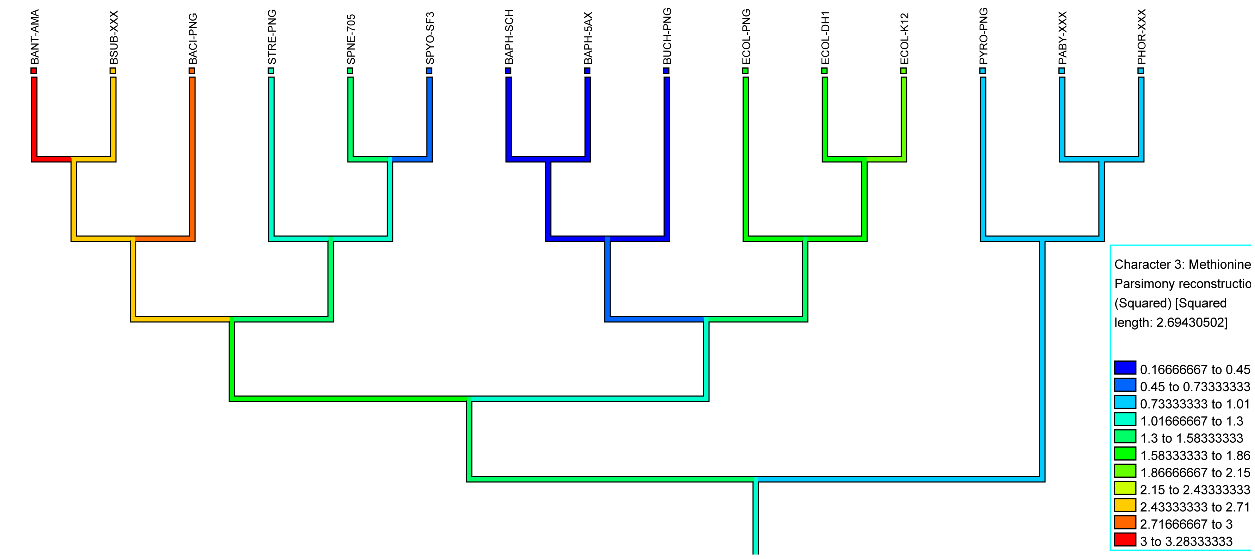
BANT-AMA
BSUB-XXX
BACI-PNG
STRE-PNG
SPNE-705
SPYO-SF3
BAPH-SCH
BAPH-5AX
BUCH-PNG
ECOL-PNG
ECOL-DH1
ECOL-K12
PYRO-PNG
PABY-XXX
PHOR-XXX
Character 3: Methionine
Parsimony reconstructio
(Squared) [Squared
length: 2.69430502]
0.16666667 to 0.45
0.45 to 0.73333333
0.73333333 to 1.01
1.01666667 to 1.3
1.3 to 1.58333333
1.58333333 to 1.86
1.86666667 to 2.15
2.15 to 2.43333333
2.43333333 to 2.71
2.71666667 to 3
3 to 3.28333333

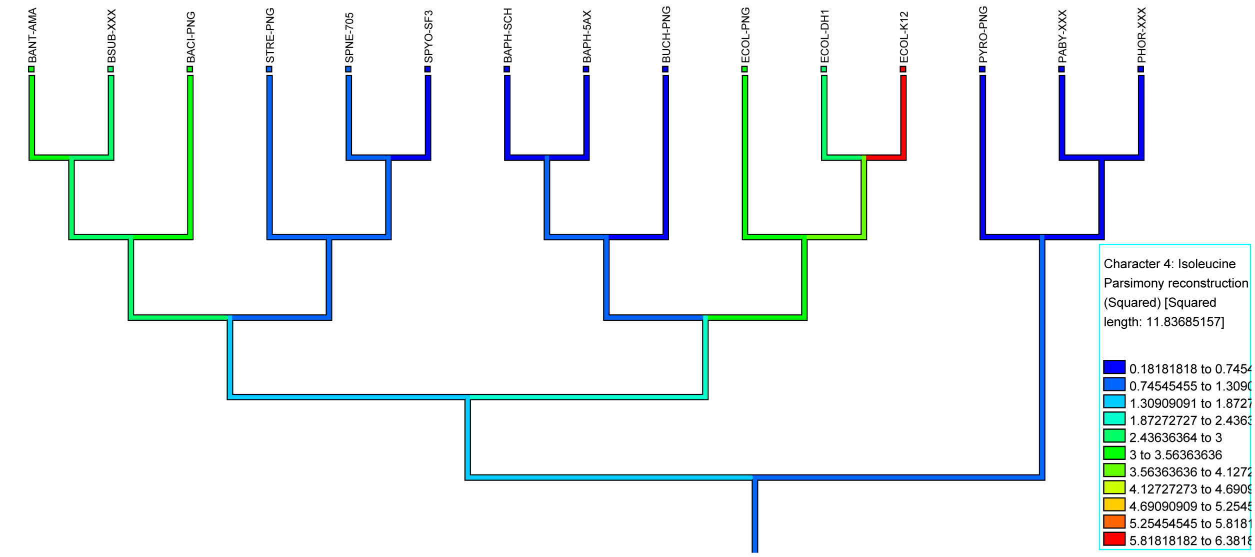
BANT-AMA
BSUB-XXX
BACI-PNG
STRE-PNG
SPNE-705
SPYO-SF3
BAPH-SCH
BAPH-5AX
BUCH-PNG
ECOL-PNG
ECOL-DH1
ECOL-K12
PYRO-PNG
PABY-XXX
PHOR-XXX
Character 4: Isoleucine
Parsimony reconstruction
(Squared) [Squared
length: 11.83685157]
0.18181818 to 0.745
0.74545455 to 1.309
1.30909091 to 1.872
1.87272727 to 2.436
2.43636364 to 3
3 to 3.56363636
3.56363636 to 4.127
4.12727273 to 4.690
4.69090909 to 5.254
5.25454545 to 5.818
5.81818182 to 6.381

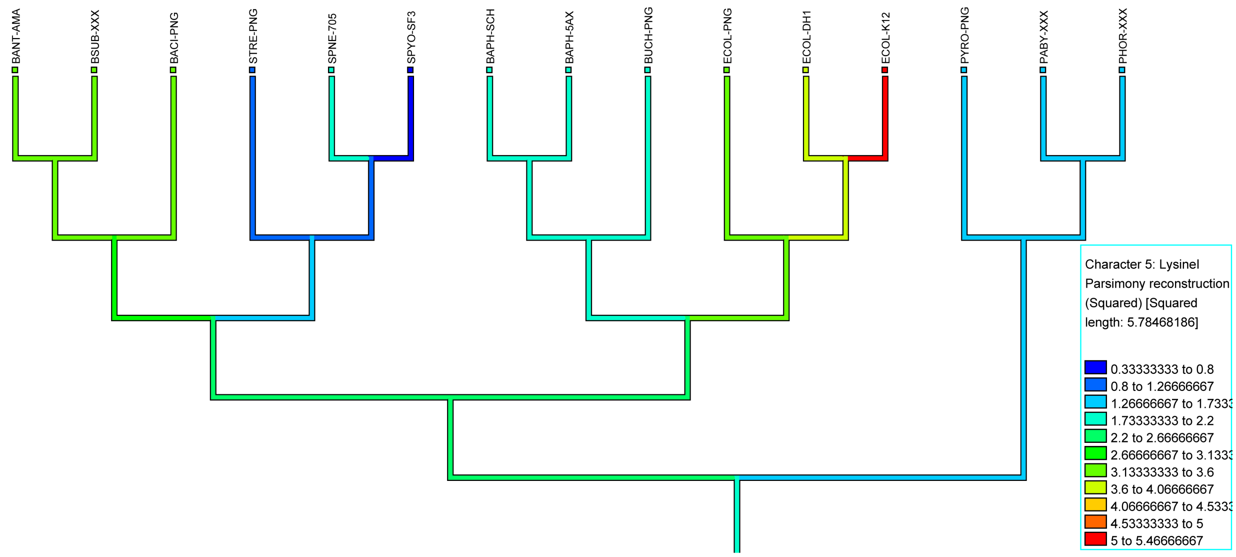
BANT-AMA
BSUB-XXX
BACI-PNG
STRE-PNG
SPNE-705
SPYO-SF3
BAPH-SCH
BAPH-5AX
BUCH-PNG
ECOL-PNG
ECOL-DH1
ECOL-K12
PYRO-PNG
PABY-XXX
PHOR-XXX
Character 5: Lysinel
Parsimony reconstruction
(Squared) [Squared
length: 5.78468186]
0.33333333 to 0.8
0.8 to 1.26666667
1.26666667 to 1.7333
1.73333333 to 2.2
2.2 to 2.66666667
2.66666667 to 3.1333
3.13333333 to 3.6
3.6 to 4.06666667
4.06666667 to 4.5333
4.53333333 to 5
5 to 5.46666667

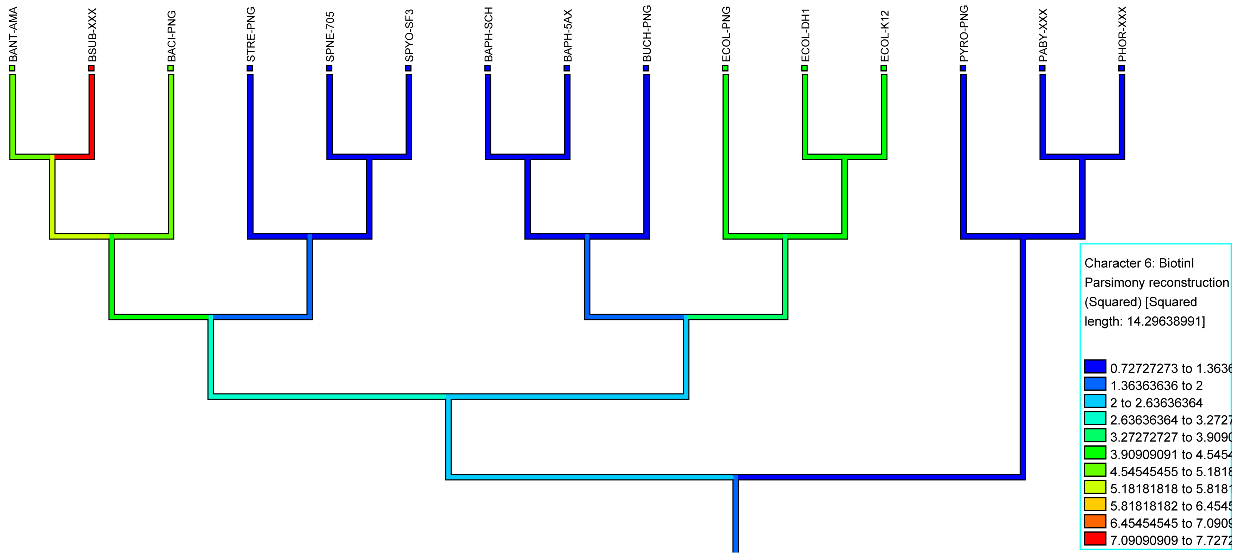
BANT-AMA
BSUB-XXX
BACI-PNG
STRE-PNG
SPNE-705
SPYO-SF3
BAPH-SCH
BAPH-5AX
BUCH-PNG
ECOL-PNG
ECOL-DH1
ECOL-K12
PYRO-PNG
PABY-XXX
PHOR-XXX
Character 6: Biotinl
Parsimony reconstruction
(Squared) [Squared
length: 14.29638991]
0.72727273 to 1.3636
1.36363636 to 2
2 to 2.63636364
2.63636364 to 3.2727
3.27272727 to 3.9090
3.90909091 to 4.5454
4.54545455 to 5.1818
5.18181818 to 5.8181
5.81818182 to 6.4545
6.45454545 to 7.0909
7.09090909 to 7.7272

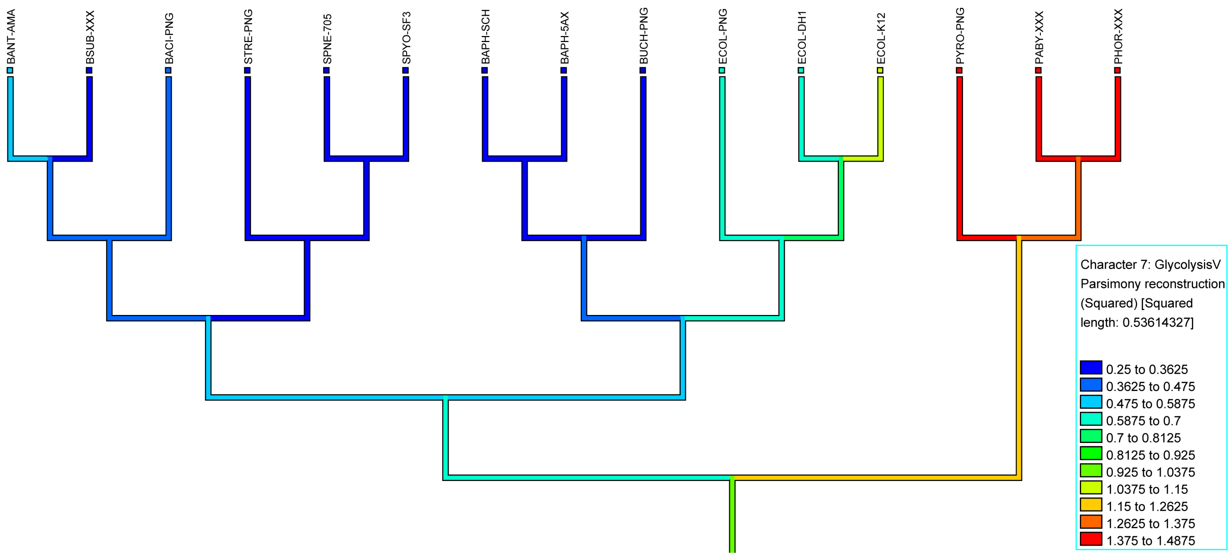
BANT-AMA
BSUB-XXX
BACI-PNG
STRE-PNG
SPNE-705
SPYO-SF3
BAPH-SCH
BAPH-5AX
BUCH-PNG
ECOL-PNG
ECOL-DH1
ECOL-K12
PYRO-PNG
PABY-XXX
PHOR-XXX
Character 7: GlycolysisV
Parsimony reconstruction
(Squared) [Squared
length: 0.53614327]
0.25 to 0.3625
0.3625 to 0.475
0.475 to 0.5875
0.5875 to 0.7
0.7 to 0.8125
0.8125 to 0.925
0.925 to 1.0375
1.0375 to 1.15
1.15 to 1.2625
1.2625 to 1.375
1.375 to 1.4875

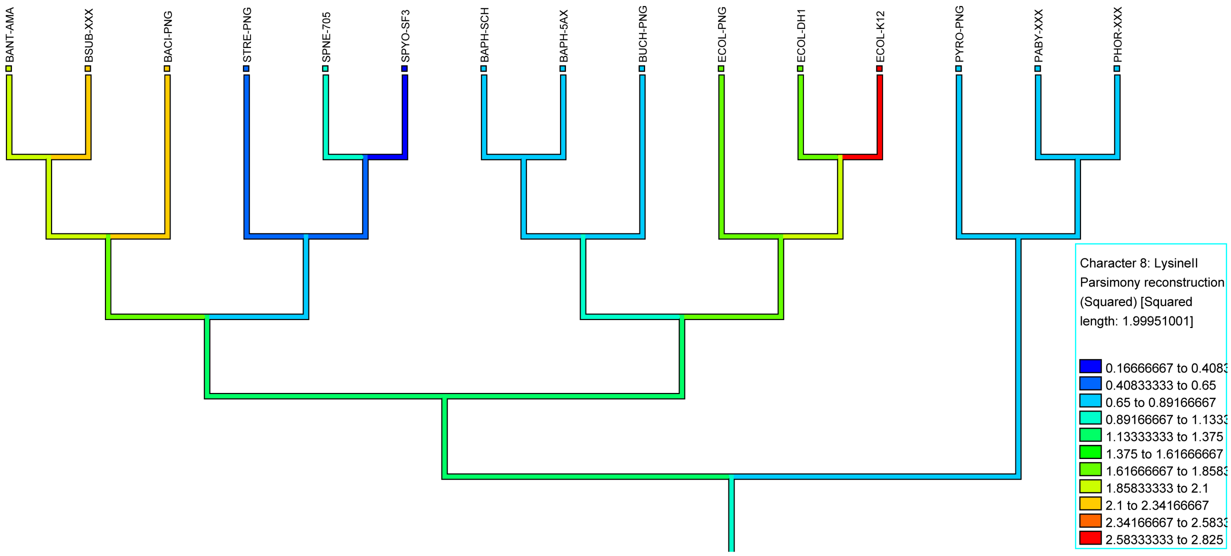
BANT-AMA
BSUB-XXX
BACI-PNG
STRE-PNG
SPNE-705
SPYO-SF3
BAPH-SCH
BAPH-5AX
BUCH-PNG
ECOL-PNG
ECOL-DH1
ECOL-K12
PYRO-PNG
PABY-XXX
PHOR-XXX
Character 8: LysineII
Parsimony reconstruction
(Squared) [Squared
length: 1.99951001]
0.16666667 to 0.4083
0.40833333 to 0.65
0.65 to 0.89166667
0.89166667 to 1.1333
1.13333333 to 1.375
1.375 to 1.61666667
1.61666667 to 1.8583
1.85833333 to 2.1
2.1 to 2.34166667
2.34166667 to 2.5833
2.58333333 to 2.825

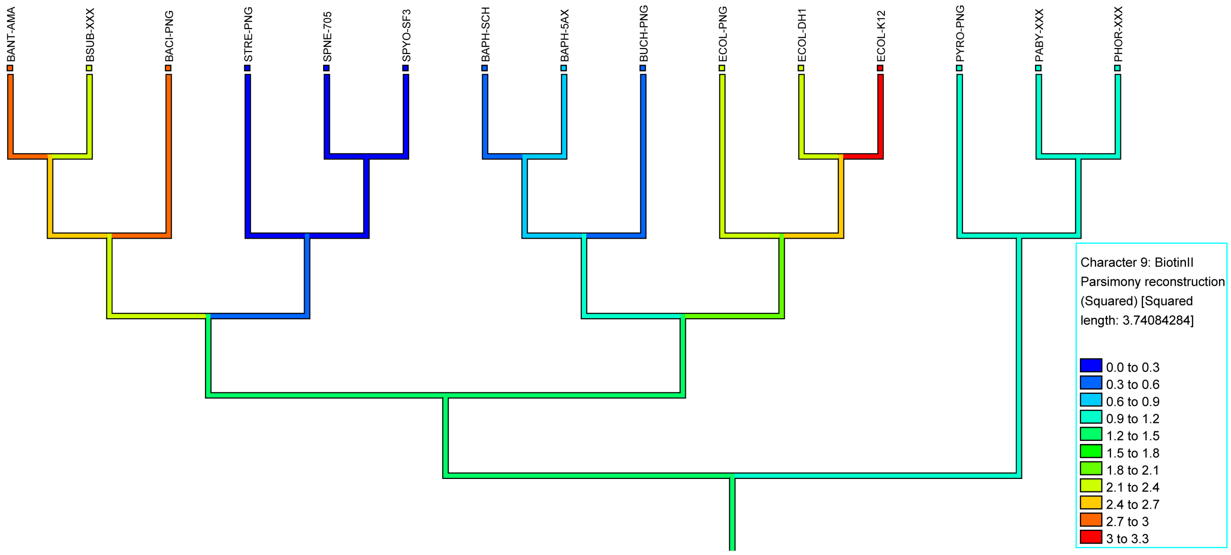

Figshare repository: data sources

- Sequence file in `FASTA` format of the 86 query enzymes corresponding to the nine pathways used in this study – DOI: 10.6084/m9.figshare.5426710.v1.
- Genome collection (as in **Table S1**) of all strains and species in pangenome ensembles used as targets in this study – DOI: 10.6084/m9.figshare.5422852.v1.
- `BLAST` output in tabular format between query enzyme sequences and target pangenome ensembles, 38,337 pairwise sequence similarities reported – DOI: 10.6084/m9.figshare.5426725.v1.
